# A stop-gain mutation in GXYLT1 promotes metastasis of colorectal cancer via the MAPK pathway

**DOI:** 10.1038/s41419-022-04844-3

**Published:** 2022-04-22

**Authors:** Lin Peng, Min Zhao, Tianqi Liu, Jiangbo Chen, Pin Gao, Lei Chen, Pu Xing, Zaozao Wang, Jiabo Di, Qiang Xu, Hong Qu, Beihai Jiang, Xiangqian Su

**Affiliations:** 1 Key laboratory of Carcinogenesis and Translational Research (Ministry of Education), Department of Gastrointestinal Surgery IV, Peking University Cancer Hospital & Institute, Beijing, 100142 China; 2 School of Science, Technology and Engineering, University of the Sunshine Coast, Maroochydore DC, QLD 4556 Australia; 3 GenomiCare Biotechnology (Shanghai) Co., Ltd, Shanghai, 201210 China; 4 Center for Bioinformatics, State Key Laboratory of Protein and Plant Gene Research, College of Life Sciences, Peking University, Beijing, 100871 P. R. China

**Keywords:** Cancer genomics, Colorectal cancer, Metastasis, Oncogenes, Cell signalling

## Abstract

Genomic instability plays a key role in the initiation and progression of colorectal cancer (CRC). Although cancer driver genes in CRC have been well characterized, identifying novel genes associated with carcinogenesis and treatment remains challenging because of tumor heterogeneity. Here, we analyzed the genomic alterations of 45 samples from CRC patients in northern China by whole-exome sequencing. In addition to the identification of six well-known CRC driver genes (*APC*, *TP53*, *KRAS*, *FBXW7*, *PIK3CA*, and *PABPC*), two tumor-related genes (*MTCH2* and *HSPA6*) were detected, along with *RRP7A* and *GXYLT1*, which have not been previously linked to cancer. *GXYLT1* was mutated in 40% (18/45) of the samples in our cohort. Functionally, GXYLT1 promoted migration and invasion in vitro and metastasis in vivo, while the GXYLT1^S212*^ mutant induced significantly greater effect. Furthermore, both GXYLT1 and GXYLT1^S212*^ interacted with ERK2. GXYLT1 induced metastasis via a mechanism involving the Notch and MAPK pathways, whereas the GXYLT1^S212*^ mutant mainly promoted metastasis by activating the MAPK pathway. We propose that GXYLT1 acts as a novel metastasis-associated driver gene and GXYLT1^S212*^ might serve as a potential indicator for therapies targeting the MAPK pathway in CRC.

## Introduction

Colorectal cancer (CRC) is the second leading cause of cancer-related death worldwide [1, 2]. Although most CRCs are regarded as sporadic diseases, the development and progression of CRC is also associated with the accumulation of genetic aberrations and mutations in tumor-suppressor genes and oncogenes [3].

Many recent studies illustrate the genomic landscape underlying the carcinogenesis of CRC [4–9]. A genetic study by The Cancer Genome Atlas (TCGA) profiled genomic changes in CRC and identified common somatic mutations in several genes critical in CRC development [6]. These common mutations include mutations in *APC*, *TP53*, *SMAD4*, *PIK3CA*, and *KRAS*, which are commonly observed in various cancers, as well as frequent mutations in *ARID1A*, *SOX9*, and *FAM123B/WTX*. Moreover, the components of several pathways were found to have hypermutation status and recurrent alterations in CRC [10, 11].

Recent studies have indicated that the frequency of gene mutation and altered genetic events are different in CRC patients from different populations. Whole-exome sequencing (WES) on African-American CRC patients identified significant distinct mutational landscapes, which suggests different disease mechanisms in patients from diverse ethnic backgrounds [12]. Furthermore, analysis of the genomic landscape in Japanese CRC patients revealed different somatic gene mutations and mutation frequencies compared with those in Caucasian patients [13]. Consistently, the frequency and sequence of gene mutations are different between Chinese CRC patients and those in other countries [14]. Moreover, genomic alterations of Chinese CRC patients show considerable heterogeneity across different regions [14–17]. Therefore, exploring the genetic events in CRC Chinese patients from different regions may improve our understanding of the somatic mutations involved in CRC progression.

In this study, we aimed to investigate the genomic landscape of patients with CRC in northern China to identify novel mutations and corresponding driver genes associated with CRC development. WES was performed in 45 patients with CRC to determine genomic alterations. Our results identified *GXYLT1* as a novel cancer-related gene with the ability to promote metastasis in CRC cells, and the stop-gain mutant GXYLT1^S212*^ mainly enhanced metastasis through MAPK pathway activation. These findings suggest that the GXYLT1^S212*^ mutant may serve as a biomarker for MAPK pathway-targeting treatments in CRC patients.

## Materials and methods

### Patients

This study included 45 CRC patients who underwent CRC resection from 2015 to 2016 at Peking University Cancer Hospital & Institute (Beijing, China) and were pathologically confirmed by two pathologists. Clinical stage was determined according to the eighth staging system of the American Joint Committee on Cancer. Patient clinicopathological characteristics are listed in Supplementary Tables 1 and 2. Written informed consent was obtained from each patient before fresh cancer tissues and peripheral blood were obtained for WES. This study was approved and supervised by the Research Ethics Committee of Peking University Cancer Hospital & Institute (No. 2014KT97).

### Whole-exome sequencing

For blood samples and cancer samples with sufficient high-quality DNA, we carried out molecular biological tests to determine whether the two DNAs were matched and from the same patient. After bacterial and viral contamination had been removed, DNA samples were sheared using a Covaris S220 sonicator and libraries were prepared using an Agilent SureSelect Human All Exon v7 kit (cat #5991–9039, Agilent, Wilmington, DE, USA), according to the manufacturer’s protocols. DNA fragments of 200 bp were sequenced using 150bp paired-end reads with a NovaSeq-6000 (Illumina), achieving a mean depth of ≥200× for tumor DNA and ≥100× for normal DNA.

### Mutational signature analysis

To explore the overall mutational features in our dataset, we focused on single-nucleotide mutations and calculated the frequency of the six substitution patterns. Based on a comprehensive analysis of COSMIC data [18] and the COSMIC mutational signature analysis pipeline, each substitution pattern was further separated into 16 substitution categories based on the flanking nucleotides surrounding the mutated base. A data matrix was constructed to represent each of the 45 exome-sequenced samples (columns) and the proportion of the 96 substitution categories (rows). Using the R package “MutationalPatterns” [19], we applied the nonnegative matrix factorization (NMF) algorithm to our data matrix and identified four mutational signatures in our 45 samples. By overlapping the 30 previously identified mutational signatures from COSMIC, we evaluated the associations between our four mutational signatures and the 30 known signatures by calculating the cosine similarity.

### Cell lines, cell culture, and reagents

Human CRC cell lines (RKO, LoVo, HCT116, and SW480) were purchased from the American Type Culture Collection (ATCC, Manassas, VA, USA), recently been authenticated by STR, and tested mycoplasma negative. Cells were cultured in RPMI-1640 or DMEM medium (HyClone, Logan, UT, USA) supplemented with 10% FBS, penicillin (100 units/mL), and streptomycin (100 µg/mL) in a humidified incubator with 5% CO_2_ at 37 °C. The Notch inhibitor DAPT (Cat #S2215) and ERK inhibitor SCH772984 (Cat #S7101) were purchased from Selleck Chemicals (Houston, TX, USA).

### In vivo metastasis assays

All animal experiments were approved by the Ethics Committee of the Peking University Cancer Hospital & Institute and performed in accordance with experimental animal management ordinance. Female BALB/c nude mice (6–8 weeks old) were purchased from the Hua-Fu-Kang Corporation (Beijing, China), randomly divided into three groups with comparable body weight (*n* = 5 per group), and housed under specific pathogen-free conditions. After anesthetizing the mice using 1.2% avertin, their abdominal cavities were opened and the spleens were injected with 5 × 10^6^ HCT116 cells stably expressing wild-type (WT) GXYLT1 or the mutant GXYLT1^S212*^. Bioluminescence imaging was conducted using an IVIS (PerkinElmer, Hopkinton, MA, USA), and image radiance values were normalized using Living Image software (PerkinElmer). After four weeks, mice were sacrificed and the livers with metastasis were isolated, fixed with 4% paraformaldehyde, and metastatic nodules counted in a single-blinded manner. The tissues were then embedded in paraffin and stained with hematoxylin and eosin (H&E) for histological analysis.

### Statistical analyses

The human CRC gene expression profiles used in this study were downloaded from the Gene Expression Omnibus (GEO, http://www.ncbi.nlm.nih.gov/geo/) and The Cancer Genome Atlas (TCGA, https://portal.gdc.cancer.gov/). The disease-free survival and overall survival of CRC patients were determined using Kaplan–Meier survival analysis and the log-rank test. GSEA was performed to evaluate the mRNA levels of GXYLT1 with associated pathways using GSEA software 4.1.0. Pearson correlation analysis was used to evaluate the association between mRNA expressions of EGFR, ERK2, and GXYLT1. Two-tailed Student’s t-tests were performed to compare two different groups, while one-way ANOVA was used to compare more than two groups. Data are presented as the mean ± standard deviation (SD) of at least three independent experiments. All statistical analyses were performed using SPSS 23.0 software (SPSS Inc., Chicago, IL) or GraphPad Prism 8 software (GraphPad Prism, La Jolla, CA, USA). Statistical significance was set at *p-*value <0.05.

Additional materials and methods are provided in Supplementary materials and methods.

## Results

### Mutation overview and tumor mutational burden (TMB) analysis in 45 CRC samples

**Fig. 1:**
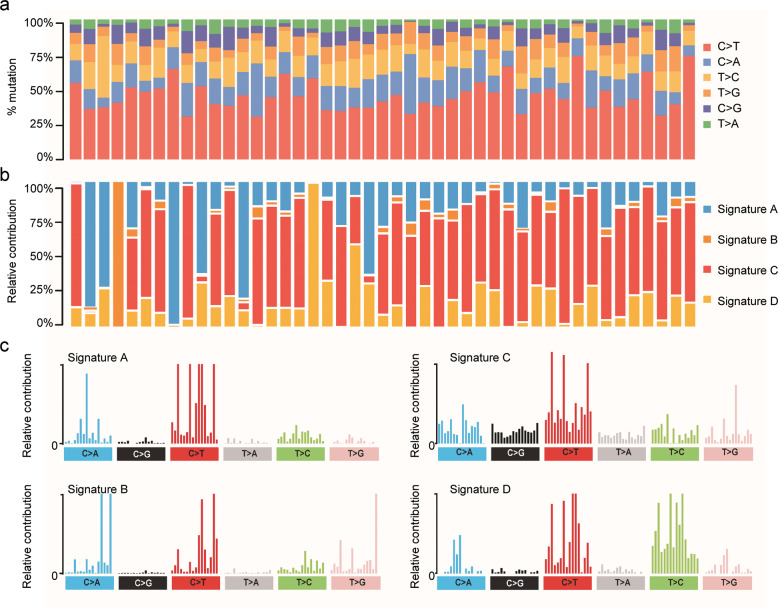
Somatic mutational signature analyses. **a** Proportional contribution of six base-substitution patterns in each sample. **b** Relative contribution of the four mutational signatures in individual patients. **c** Patterns of the four mutational signatures A–D in 45 CRC samples.

We first performed WES on tumors and paired blood samples from 45 Chinese patients with CRC and obtained approximately 460 Gb of read data with an average sequencing depth of 200-fold for tumors and 100-fold for blood samples. Further mapping to the human reference genome identified 22472 somatic single-nucleotide variants (SNVs), which were classified as missense, nonsense, frameshift, splice site, translation start site, and nonstop mutations (Supplementary Fig. 1a and Table 3). Moreover, 1063 short insertions and 4005 short deletions were detected in our cohort (Supplementary Fig. 1b and Table 3). Mutational signature analysis revealed that transition mutations were more common than transversion mutations (Supplementary Fig. 1c), and C > T substitutions were predominant in our samples (Fig. 1a and Supplementary Fig. 1d).

To examine the overall genomic features of our data, we compared 33 publicly available TCGA cancer mutational datasets using TMB analysis. The TMB of our data was similar to that of TCGA COAD (CRC) dataset, suggesting that our data had a similar mutational burden to the well-established CRC cohort (Supplementary Fig. 2).

### Identification of four mutational signatures in our cohort

A previous signature analysis of all cancer mutation data from the COSMIC database linked different cancer types to different contributions from each mutational spectrum [18]. To better understand the pathogenesis of CRC, we performed mutational signature analysis on all 22472 SNVs by analyzing the six mutation classes (C > T, C > A, T > C, T > G, C > G, and T > A). We identified four mutational signatures in our CRC samples (Signatures A–D) based on the calculated frequency of the six substitution patterns (Fig. 1b, c). Moreover, the relative contributions of these four signatures, which are the counterparts of the contributions observed for the 30 COSMIC signatures, varied among the 45 samples (Fig. 1b and Supplementary Fig. 3a).

Signature A was characterized by dominant C > A substitution with 92% similarity to COSMIC Signature 6 (Fig. 1c and Supplementary Fig. 3b), which is the most common signature in CRC, and this signature was detected in less than 3% of examined samples of other cancer types [18]. Samples with Signature 6 are generally microsatellite-unstable tumors associated with defective DNA mismatch repair.

Signature B in our cohort displayed a unique C > A feature in TpCpT and T > G in TpTpT (Fig. 1c), with 98% similarity to COSMIC Signature 10 (Supplementary Fig. 3b), which is characterized by large numbers of mutations in small subsets of samples, notably colorectal and uterine cancers [18]. Therefore, tumor samples exhibiting this mutational signature are often grouped as ultrahypermutators.

Signature C, which was characterized by mutations in all six substitution categories, was a predominant signature in our 45 CRC samples with 80% similarity to COSMIC Signature 1 (Fig. 1c and Supplementary Fig. 3b), which is the most commonly reported signature in all cancer types including CRC.

Signature D exhibited a uniquely high proportion of T > C substitution (Fig. 1c), similar to COSMIC Signature 6 (cosine similarity = 0.81) (Supplementary Fig. 3b). Together, our mutational analysis identified some well-known CRC signatures and confirmed the results of our TMB analysis, suggesting that our data have similar signature features to known CRC cohorts.

### Driver mutation analysis revealed *GXYLT1* as a potential driver gene in CRC

**Fig. 2:**
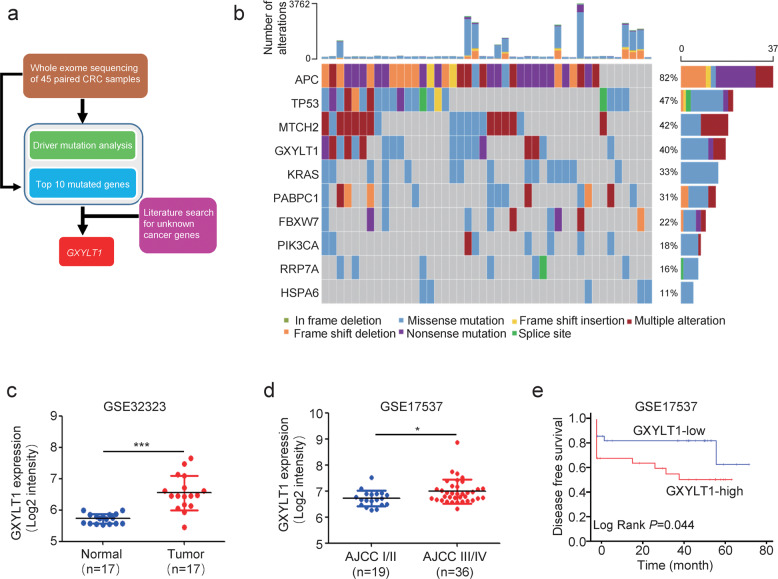
Somatic alterations and driver gene analysis in 45 CRC samples. **a** Schematic of the workflow for the identification of novel driver genes. **b** Sample-based OncoPrint of the top- 10 driver genes identified using the IntOGen algorithm. Each column represents an individual sample, and each row represents a gene. Top: total number of somatic alterations (*y* axis) in each sample (*x* axis). Right: percentage of somatic alterations in 45 CRC samples. **c** Analysis of *GXYLT1* mRNA expression levels in CRC tumors and matched normal tissues (GSE32323). **d** Analysis of *GXYLT1* mRNA expression levels with respect to tumor stage (GSE17537). **e** Kaplan–Meier survival analysis of *GXYLT1* levels and survival (GSE17537) in GEO datasets. *p < 0.05, ***p < 0.001.

To identify novel genetic mutations in our dataset (Fig. 2a), we used the driver mutation identification pipeline IntOGen [20], which identified 495 driver mutations in 79 genes (Supplementary Table 4). Among the top-10 ranked genes in the IntOGen results from our dataset, we identified six well-studied driver genes that have been associated with cancer: *APC*, *TP53*, *KRAS*, *PABPC1*, *FBXW7*, and *PIK3DA* (Fig. 2b). We also identified four novel genes with significant OncodriveFM and OncodriveCLUST scores that have not yet been reported in CRC: *MTCH2*, *GXYLT1*, *RRP7A*, and *HSPA6*.

*MTCH2* and *HSPA6* play various roles in different cancers but have not been associated with CRC [21, 22]. Among the two genes that have not been previously linked to cancer (*GXYLT1* and *RRP7A*), *GXYLT1* was recurrently mutated in 18 of 45 samples (40%) (Fig. 2b). We therefore focused on *GXYLT1* as a novel gene potentially associated with CRC.

*GXYLT1* encodes a glucoside xylosyltransferase that contributes to the first xylose elongation of O-glucose glycans on the extracellular domain of Notch1 and Notch2 [23], which is required for the trafficking of Notch proteins to the cell surface [24]. To explore the potential role of *GXYLT1* in CRC, we first examined the mRNA levels of *GXYLT1* in GEO datasets. *GXYLT1* transcript expressions were significantly elevated in CRC tissues compared with levels in matched normal tissues from datasets GSE32323 (*p* < 0.001), GSE24550 (*p* < 0.001), and GSE9348 (*p* < 0.001) (Fig. 2c and Supplementary Fig. 4a). Moreover, *GXYLT1* mRNA levels gradually increased with tumor progression and differed significantly between tumor stages (*p* = 0.023, *p* < 0.001, and *p* = 0.056 for GSE17537, GSE33193, and GSE28702, respectively) (Fig. 2d and Supplementary Fig. 4b).

We next performed Kaplan–Meier analysis to explore the prognostic significance of *GXYLT1* expression in CRC. The results showed that patients with elevated *GXYLT1* levels had a shorter disease-free survival or overall survival than those with low *GXYLT1* levels (*p* = 0.044, *p* = 0.069, and *p* = 0.082 for GSE17537, GSE38832, and GSE17538, respectively) (Fig. 2e and Supplementary Fig. 4c). Taken together, these results suggest that elevated *GXYLT1* mRNA expression indicates a poor prognosis in CRC patients.

### GXYLT1 enhances CRC metastasis, while GXYLT1^S212*^ induces greater metastatic ability in CRC

**Fig. 3:**
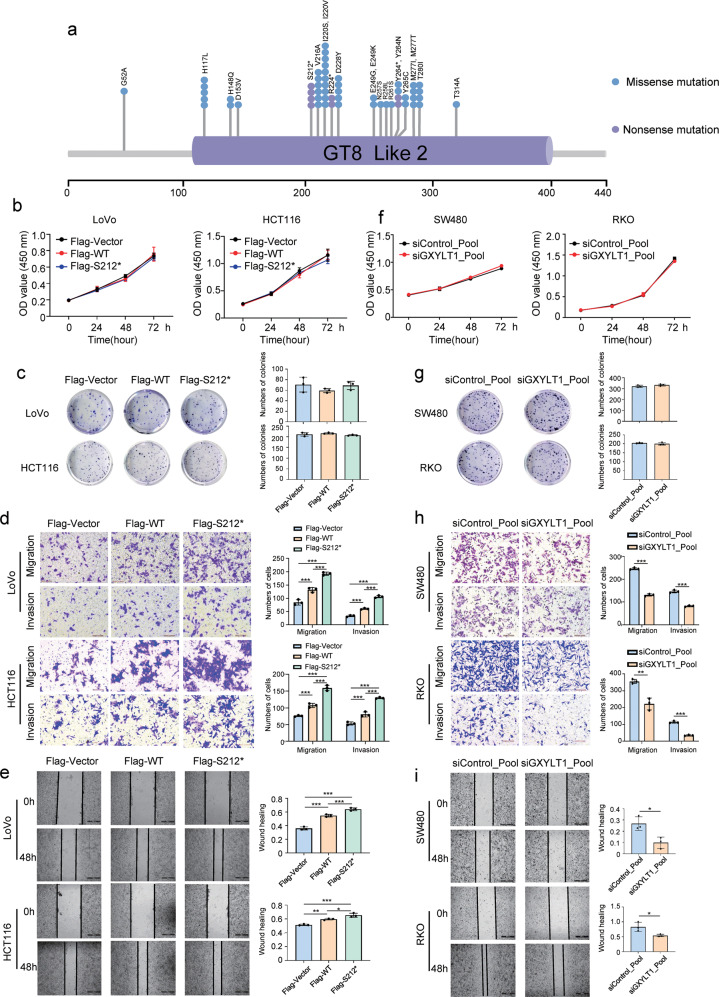
GXYLT1 promotes CRC cell migration and invasion, while GXYLT1^S212*^ exerts a stronger effect in vitro. **a** Somatic mutations in the GXYLT1 functional domain. Numbers represent amino acid residues. Each dot represents an individual mutated tumor sample. Blue dot: missense mutation. Purple dot: nonsense mutation. **b**, **f** Cell viability was assessed using CCK-8 assays in CRC cells transfected as indicated. **c**, **g** Representative images of colony formation in CRC cells transfected as indicated. Graphs show the numbers of colonies. **d**, **h** Transwell migration and invasion assays in CRC cells transfected as indicated. Graphs show quantification of migrated cells. **e**, **i** Wound-healing assays to evaluate cell migration in CRC cells transfected as indicated. Graphs show quantification of the wound-healing area. Data are presented as the mean ± SD of at least three independent experiments. *p < 0.05, **p < 0.01, ***p < 0.001.

Our results identified *GXYLT1* as a novel gene with a high mutation frequency in CRC. Twenty-two mutations were identified, most of which were located in the functional “GT8 like 2” domain of GXYLT1, including three stop-gain mutations (GXYLT1^S212*^, GXYLT1^R224*^, and GXYLT1^Y264*^) (Fig. 3a). One of the most frequently occurring mutations, GXYLT1^S212*^, was validated and recognized as a nonsense mutation in four independent samples (4/45, 8.9%) (Fig. 3a, Supplementary Fig. 5 and Table 2), compared with GXYLT1^R224*^ (2/45, 4.4%) and GXYLT1^Y264*^ (2/45, 4.4%). Thus, we focused on examining the role of GXYLT1 and mutant GXYLT1^S212*^ in CRC.

To investigate the functions of GXYLT1 and GXYLT1^S212*^ in CRC, we first examined the expression of GXYLT1 in CRC cells (Supplementary Fig. 6a, b). Since the GXYLT1 antibody is against the C-terminal 30 amino acids of the protein, GXYLT1^S212*^ could not be detected by anti-GXYLT1 (Supplementary Fig. 6c). We used the FLAG antibody to determine the overexpression levels of GXYLT1 and GXYLT1^S212*^ in CRC cells transiently transfected with GXYLT1 and GXYLT1^S212*^ plasmids (Supplementary Fig. 6d). Ectopic WT GXYLT1 expression had no significant effects on the proliferation or colony formation of CRC cells (Fig. 3b, c), but increased cell migration and invasion in the Transwell and wound-healing assays (Fig. 3d, e). Similarly, we found that GXYLT1^S212*^ had no effect on proliferation or colony-formation ability. However, mutant GXYLT1^S212*^ showed a stronger capability of promoting migration and invasion compared with WT GXYLT1 (Fig. 3d, e). We also knocked down GXYLT1 using short interfering RNA in the SW480 and RKO cells (Supplementary Fig. 6e). Consistent with the overexpression results, downregulation of GXYLT1 had no effect on CRC cell growth but attenuated CRC cell migration and invasion (Fig. 3f–i). These results indicated that GXYLT1 contributes to the migration and invasion of CRC cells and that GXYLT1^S212*^ exhibits enhanced migration and invasion-promoting ability in vitro.

**Fig. 4:**
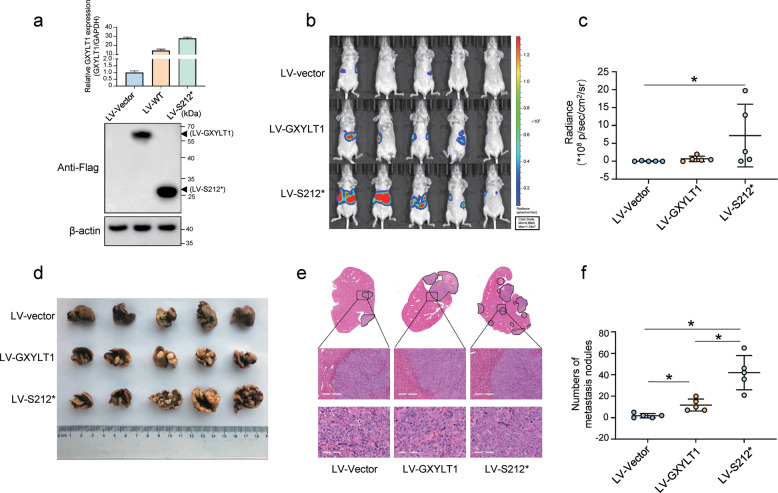
GXYLT1^S212*^ induces increased metastatic activity in CRC than WT GXYLT1 in vivo. The spleens of mice were injected with 5 × 10^6^ HCT116 cells infected with LV-control, LV-GXYLT1, or LV-S212* (*n* = 5 mice per group). **a** GXYLT1 and GXYLT1^S212*^ expressions were confirmed in HCT116 cells stably infected with LV-GXYLT1 and LV-S212*, respectively. **b** Representative bioluminescence imaging of mice two weeks after implantation. **c** Quantification of bioluminescence imaging in the mouse liver from the indicated groups. **d** Representative images of liver metastatic nodules from the indicated groups. **e** Representative images of H&E staining in liver metastatic lesions from the indicated group. Scale bars: top, 300 μm; bottom, 60 μm. **f** Number of metastatic liver nodules from the indicated groups. Data are presented as the mean ± SD (*n* = 5). *p < 0.05.

To confirm the in vitro findings, we next established LV-GXYLT1 and LV-S212* cell models (Fig. 4a). We evaluated whether WT GXYLT1 and GXYLT1^S212*^ affected CRC metastasis in vivo by injecting HCT116 cells stably overexpressing GXYLT1 or GXYLT1^S212*^ into the spleen of nude mice. Two weeks after injection, bioluminescence imaging was performed to detect tumor signals in mouse liver. Although LV-GXYLT1 showed a little increase in the metastatic potential of HCT116 cells compared with control group, LV-S212* exhibited a significant stronger effect, which was demonstrated by enhanced luminescence of the mouse liver (Fig. 4b, c). After 4 weeks, mouse livers were collected for analysis. Increased numbers of liver metastatic nodules and increased incidence of liver metastasis were observed in the LV-GXYLT1 and LV-S212* groups compared with the control group (Fig. 4d–f and Supplementary Table 5). Furthermore, the LV-S212* group had more liver metastatic nodules than the LV-GXYLT1 group (Fig. 4d–f), suggesting that LV-S212* exhibits a gain-of-function effect. Taken together, these findings suggest that GXYLT1 promotes CRC metastasis and GXYLT1^S212*^ induces a greater metastatic ability both in vitro and in vivo.

### WT GXYLT1 promotes CRC metastasis via the Notch pathway and GXYLT1^S212*^ increases metastasis partially via the Notch pathway

**Fig. 5:**
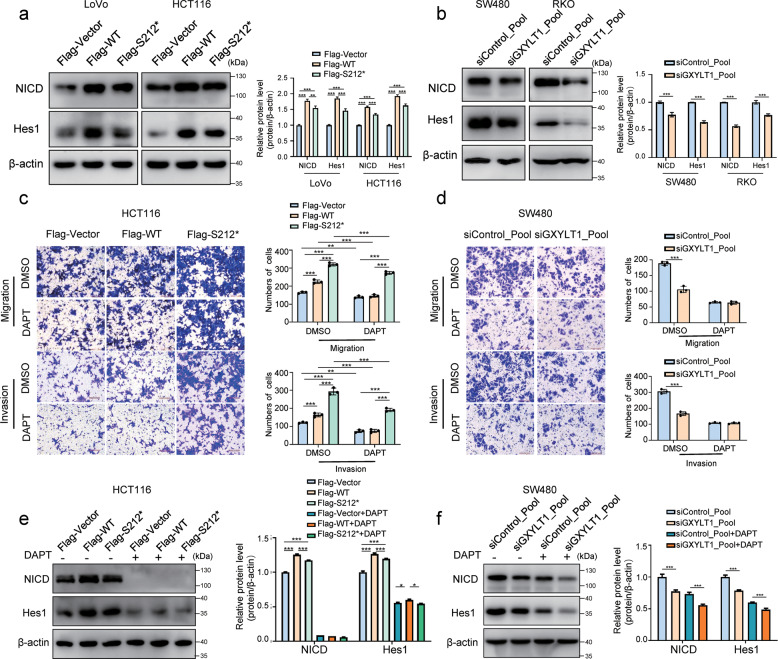
GXYLT1^S212*^ promotes migration and invasion partially via the Notch pathway, unlike Notch pathway dependence of WT GXYLT1. **a**, **b** Western blot analysis of the protein levels of the Notch protein intracellular domain (NICD) and Hes1 in CRC cells transfected as indicated. Bar graphs show quantitative analysis of protein levels. **c**, **d** Transwell migration and invasion assays of transfected CRC cells treated with the Notch pathway inhibitor DAPT or the DMSO control for 48 h. Charts show quantification of migrated cells. **e**, **f** Western blot analysis of NICD and Hes1 in cell lysates. Bar graphs show quantification of NICD and Hes1 protein levels. Data are presented as the mean ± SD of at least three independent experiments. *p < 0.05, **p < 0.01, ***p < 0.001.

To investigate whether GXYLT1 regulates the Notch pathway in CRC cells, we examined the protein levels of the intracellular domain of the Notch protein (NICD) and Hes1, which is encoded by one of the major Notch-targeted genes. Overexpression of WT GXYLT1 and GXYLT1^S212*^ in CRC cells upregulated NICD and Hes1 levels (Fig. 5a), indicating activation of the Notch pathway. Notably, GXYLT1^S212*^ induced NICD and Hes1 expression to lower levels than those induced by WT GXYLT1. Consistently, GXYLT1 knockdown decreased NICD and Hes1 expression in SW480 and RKO cells (Fig. 5b). These results indicated that GXYLT1 regulates the Notch pathway in CRC cells. Furthermore, GXYLT1^S212*^ promoted the Notch pathway cascade to a lower level compared with WT GXYLT1.

To determine whether WT GXYLT1 and GXYLT1^S212*^ promote CRC cell metastasis via the Notch pathway, we abolished Notch pathway activity using the Notch inhibitor, DAPT. Transwell assays demonstrated that treatment of CRC cells with DAPT significantly eliminated the induction of cell migration and invasion by WT GXYLT1, but had a modest effect on cells expressing GXYLT1^S212*^ (Fig. 5c and Supplementary Fig. 7a). DAPT treatment also inhibited cell migration and invasion in both GXYLT1-depleted and control cells (Fig. 5d and Supplementary Fig. 7b). Moreover, DAPT markedly decreased NICD and Hes1 protein levels in WT GXYLT1- and GXYLT1^S212*^-overexpressing cells, as well as in GXYLT1-knockdown cells (Fig. 5e, f and Supplementary Fig. 7c, d). These results suggest that Notch pathway inhibition prevents cell migration and invasion induced by WT GXYLT1 but not GXYLT1^S212*^. Therefore, these data raise the possibility that GXYLT1^S212*^ promotes CRC metastasis only partially through the Notch pathway, and that alternative pathways are potentially involved in the induction of metastasis by GXYLT1^S212*^.

### GXYLT1^S212*^ enhances metastasis mainly via the MAPK pathway

**Fig. 6:**
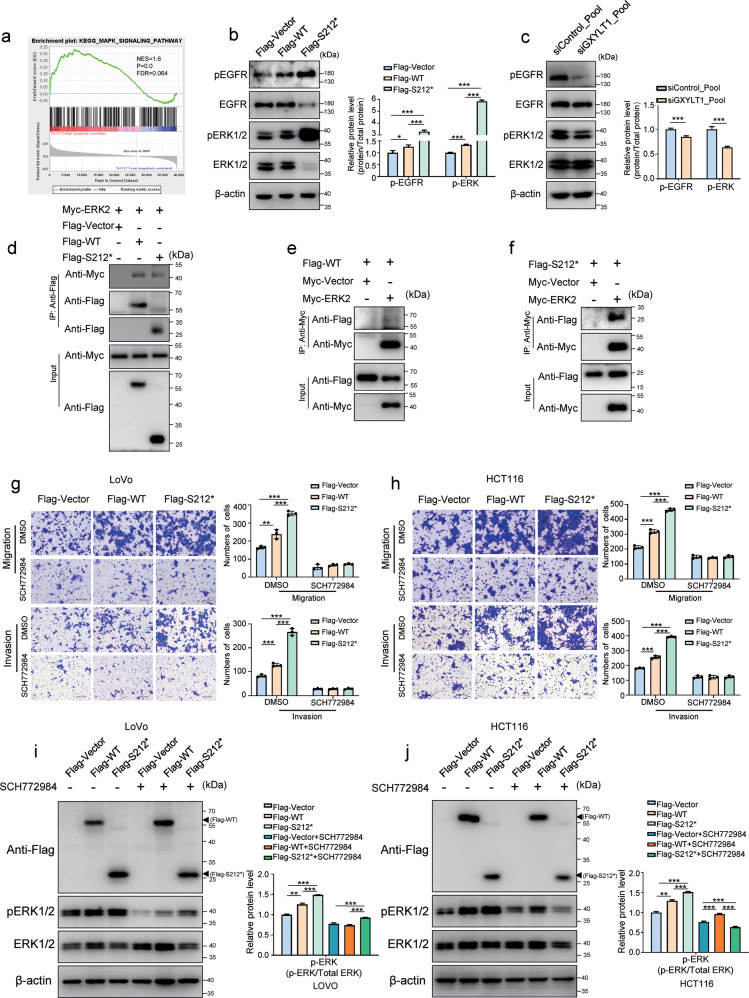
GXYLT1^S212*^ activates the MAPK pathway to promote migration and invasion in CRC cells. **a** GSEA analysis of TCGA dataset revealed the correlation between GXYLT1 expression and the MAPK pathway. **b**, **c** Western blot analysis of protein levels and phosphorylation levels of EGFR and ERK1/2 in transfected CRC cells. Bar graphs show quantification of pEGFR and pERK1/2 levels. **d**–**f** Exogenous GXYLT1 and GXYLT1^S212*^ interact with EKR2. Co-immunoprecipitation was performed in cells transfected as indicated using Flag antibody to pull down Flag-GXYLT1 and Flag-GXYLT1^S212*^ (**d**) or Myc antibody against Myc-ERK2 (**e**, **f**). Western blot was performed using the indicated antibodies. **g**, **h** Transwell migration and invasion assays of CRC cells expressing WT GXYLT1 or GXYLT1^S212*^ and treated with the ERK1/2 inhibitor SCH772984 or the DMSO control for 48 h. Graphs show quantification of migrated cells. **i**, **j** Western blot analysis of cell lysates with the indicated antibodies. Bar graphs show quantification of pERK1/2 levels. Data are presented as the mean ± SD of at least three independent experiments. *p < 0.05, **p < 0.01, ***p < 0.001.

Accumulating evidence has indicated that the interactions between the Notch and MAPK pathways are associated with the progression of CRC [25, 26]. Gene set enrichment analysis (GSEA) of the TCGA dataset suggested that GXYLT1 expression was positively associated with MAPK pathway (Fig. 6a). Furthermore, Pearson correlation analyses of the public CRC databases indicated that mRNA levels of GXYLT1 were positively correlated with EGFR and ERK2 (Supplementary Fig. 8). Thus, we next investigated whether WT GXYLT1 and GXYLT1^S212*^ regulate the MAPK pathway in CRC cells by detecting the phosphorylation levels of EGFR and ERK1/2. Both WT GXYLT1 and GXYLT1^S212*^ overexpression enhanced the phosphorylation of EGFR and ERK1/2 in LoVo cells (Fig. 6b). In addition, we observed that GXYLT1^S212*^ enhanced EGFR and ERK1/2 phosphorylation to greater levels compared with those observed in cells expressing WT GXYLT1. Consistently, depletion of GXYLT1 resulted in decreased phosphorylation of EGFR and ERK1/2 in RKO cells (Fig. 6c). These results suggested that compared with WT GXYLT1, GXYLT1^S212*^ strongly promoted the MAPK pathway.

To further explore the relationship between GXYLT1 or GXYLT1^S212*^ and ERK1/2, we performed co-immunoprecipitation. Myc-ERK2 was found in Flag-GXYLT1 and Flag-GXYLT1^S212*^ immune complexes (Fig. 6d), while Flag-GXYLT1 and Flag-GXYLT1^S212*^ were detected in Myc-ERK2 immunoprecipitates through reciprocal immunoprecipitation (Fig. 6e, f). These findings suggest that GXYLT1 and GXYLT1^S212*^ interact with ERK2 in vivo.

To evaluate whether WT GXYLT1 and GXYLT1^S212*^ enhanced cell metastasis via the MAPK pathway, we inhibited MAPK pathway activity using the ERK kinase inhibitor SCH772984 and analyzed the migration and invasion of CRC cells expressing WT GXYLT1 or GXYLT1^S212*^. SCH772984 significantly blocked migration and invasion (Fig. 6g, h) and suppressed ERK1/2 phosphorylation (Fig. 6i, j) induced by WT GXYLT1 and GXYLT1^S212*^ in CRC cells.

Taken together, our results demonstrated that both WT GXYLT1 and GXYLT1^S212*^ enhance CRC metastasis via the MAPK pathway by interacting with ERK, and GXYLT1^S212*^ had stronger promoting effects compared with WT GXYLT1.

### GXYLT1 and GXYLT1^S212*^ are required for the activities of Notch and MAPK pathways

**Fig. 7:**
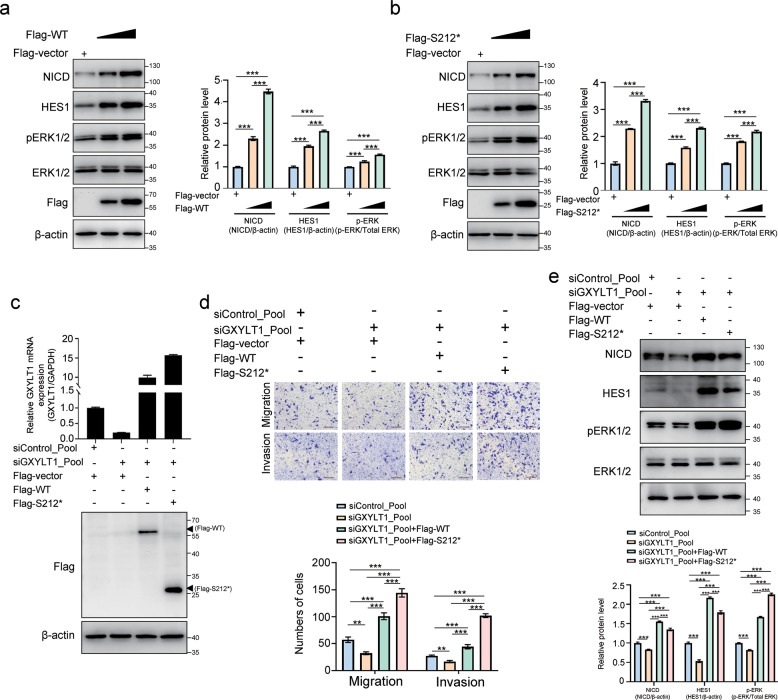
GXYLT1 and GXYLT1^S212*^ are required for the activities of Notch and MAPK pathways. **a**, **b** Western blot analysis of cell lysates with the indicated antibodies. LoVo cells were transfected with different amounts of GXYLT1 and GXYLT1^S212*^ expression plasmid and harvested for western blot after 48 h. Bar graphs show quantification of NICD, Hes1, and pERK1/2 levels. **c** RKO cells with indicated transfection were harvested for RT-PCR analysis and Western blot analysis to determine the expression of GXYLT1 and GXYLT1^S212*^. **d** Transwell migration and invasion assays of RKO cells with indicated transfection. Graphs show quantification of migrated cells. **e** Western blot analysis of cell lysates with the indicated antibodies. RKO cells with indicated transfection were harvested for Western blot after 48 h. Bar graphs show quantification of NICD, Hes1, and pERK1/2 levels. Data are presented as the mean ± SD of at least three independent experiments. *p < 0.05, **p < 0.01, ***p < 0.001.

To further clarify whether the activities of Notch and MAPK pathways are dependent on GXYLT1 and GXYLT1^S212*^, LoVo cells were transfected with the GXYLT1 and GXYLT1^S212*^ plasmid in a dose-dependent manner, and the endogenous expression of NICD, Hes1, and pERK1/2 was determined. The gradual increase in NICD, Hes1, and pERK1/2 levels was observed post GXYLT1 or GXYLT1^S212*^ dose-dependent transfection (Fig. 7a, b).

Furthermore, rescue experiments were performed in GXYLT1-depleted RKO cells transfected with Flag-GXYLT1 and Flag-S212* (Fig. 7c). Transwell assays showed that WT GXYLT1 and GXYLT1^S212*^ significantly rescued the migration and invasion deficit induced by GXYLT1 knockdown in RKO cells, and GXYLT1^S212*^ had a stronger effect (Fig. 7d). Moreover, restoring the expression of WT GXYLT1 and GXYLT1^S212*^ rescued the GXYLT1 depletion-induced downregulation of NICD, Hes1, and pERK1/2 (Fig. 7e), which are effectors of the Notch and MAPK pathway. These results suggested that Notch and MAPK signaling are downstream of GXYLT1 and GXYLT1^S212*^.

## Discussion

In this study, we performed WES on samples from 45 patients with CRC in northern China to identify genomic alterations and potential genomic targets for clinical diagnosis and treatment. We identified four mutation signatures in our cohort with a high prevalence of signature C. Our combined computational prediction-based prioritization and functional analysis identified that GXYLT1 is a potentially novel oncogene that promotes the metastasis of CRC via the Notch and MAPK pathways, and the stop-gain mutant GXYLT1^S212*^ promoted a stronger malignant phenotype in CRC by activating MAPK signaling.

Increasing evidence has shown a high rate of C > T transitions in diverse types of cancers including CRC [27–30]. Consistent with previous studies, C > T substitutions were predominant in our cohort. In addition, the TMB in our cohort was similar to that of TCGA dataset, indicating a comparable mutational frequency in the protein-coding regions. Moreover, mutational signature analysis revealed four prominent mutational patterns in our CRC cohort. Signatures A and D were similar to the COSMIC Signature 6, while Signature B and C corresponded to COSMIC Signature 10 and 1, respectively.

Gene mutations, such as those in *APC*, *KRAS*, and *TP53*, have been established as core elements that facilitate the malignant transformation of CRC cells [6, 8]. Similarly, we found that *APC* (82%), *TP53* (47%), *KRAS* (33%), *PABPC1* (31%), *FBXW7* (22%), and *PIK3CA* (18%) were mutated at a high frequency in our cohort. In addition, we identified previously uncharacterized mutated genes in CRC, such as *MTCH2* (42%), *GXYLT1* (40%), *RRP7A* (16%), and *HSPA6* (11%).

In addition to *APC*, *TP53*, and *MTCH2*, *GXYLT1* displayed a high frequency of somatic mutations (40%) in our cohort. However, mutations in *GXYLT1* have only been detected in 0.9‒2% of CRC samples from TCGA and other published cohorts [6, 8, 27]. The differences between our study and previous studies may be from substantial differences in the epidemiological characteristics of the patient cohorts.

Gene mutations play critical roles in the tumorigenesis and metastasis of CRC [31]. Previous studies showed that stop-gain mutations of *APC* deactivate the tumor-suppressor functions of the encoded proteins and confer oncogenic gain-of-function activity, resulting in the rapid development of aggressive carcinoma [32–34]. Moreover, the truncated PPM1D induced by exon mutation impairs the activation of p53 pathway, and promotes tumor growth in Apc^min^ mice, compared with the wild-type PPM1D [35]. Our study identified new recurrent alterations in GXYLT1, three of which were stop-gain mutations (GXYLT1^S212*^, GXYLT1^R224*^, and GXYLT1^Y264*^). Thus, GXYLT1^S212*^ and GXYLT1^Y264*^ with relatively short and long amino acid sequences, respectively, were selected to explore their roles in CRC. The results showed that the GXYLT1^S212*^ exhibited significantly increased migration and invasion ability compared with WT GXYLT1. However, no significant effects were observed for the GXYLT1^Y264*^ (Supplementary Fig. 9). Therefore, GXYLT1^S212*^ was chosen for mechanistic studies.

Although the Notch pathway has been associated with CRC development and progression [36–38], the role of xylosylation in the Notch ECD remains controversial. Xylose modification of Notch by GXYLT1 and GXYLT2 suppresses Notch activity in *Drosophila* [39, 40], whereas xylosylation by GXYLT2 upregulates the Notch pathway in human cancers [41]. Since there is no significant association between the expression of GXYLT1 and GXYLT2 in available databases (Supplementary Fig. 10), we investigated the functions of WT GXYLT1 and GXYLT1^S212*^. Consistent with a previous study on GXYLT2, our results showed that the Notch signaling pathway is required for WT GXYLT1-regulated metastasis. However, GXYLT1^S212*^ increased the metastatic ability of CRC cells with relatively lower Notch pathway activation compared with WT GXYLT1. Despite the stronger metastatic ability induced by GXYLT1^S212*^, these effects were not completely blocked by the Notch inhibitor DAPT, indicating that other mechanisms may be involved in the effects of GXYLT1^S212*^ on CRC metastasis.

**Fig. 8:**
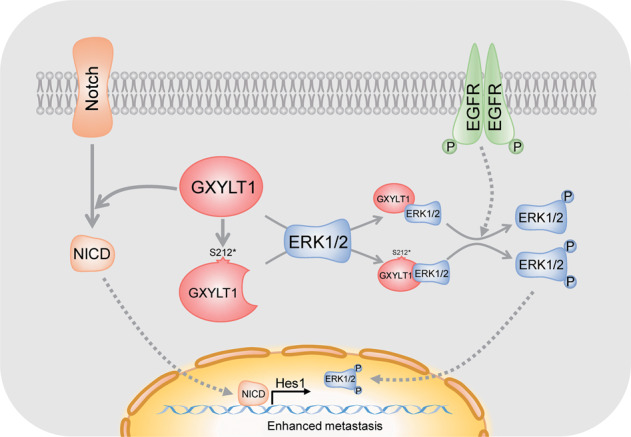
A schematic model illustrates the potential mechanisms by which GXYLT1 and GXYLT1^S212*^ promote the metastasis of CRC. GXYLT1 promoted CRC metastasis via the Notch and MAPK pathways, whereas GXYLT1^S212*^ exhibited stronger metastasis-promoting ability mainly through activating the MAPK pathway.

Research has demonstrated that the crosstalk between the MAPK and Notch pathways is involved in cancer progression [25, 26]. MAPK pathway activation promotes Notch signaling [42, 43], whereas its inhibition suppresses Notch signaling by decreasing NICD [44] and Hes1 [45] expression. Therefore, we evaluated the effects of WT GXYLT1 and GXYLT1^S212*^ on the MAPK signaling pathway. Our results showed that both WT GXYLT1 and GXYLT1^S212*^ interact with ERK2, and GXYLT1^S212*^ induced a greater effect on the MAPK cascade than WT GXYLT1 (Fig. 8). Inhibiting the MAPK pathway completely abrogated the promotion of CRC cell metastasis by WT GXYLT1 and GXYLT1^S212*^. Since the Notch pathway inhibitor only partially repressed the metastasis induced by GXYLT1^S212*^, we hypothesize that GXYLT1^S212*^ mainly enhances metastasis via the MAPK signaling pathway. Together, these data suggest that ERK1/2 inhibitors could potentiate CRC therapies targeting GXYLT1 in the presence of mutant GXYLT1.

The roles of WT GXYLT1 and GXYLT1^S212*^ in CRC metastasis of our study should be interpreted with some considerations. First, our patients are from northern China, and this mutation pattern may be unique to CRC patients from northern China. Second, a larger cohort of patients with CRC, including detailed clinicopathological information, is required to validate the clinical significance of WT GXYLT1 and GXYLT1^S212*^ in patients with CRC. Third, although the ERK1/2 inhibitor completely abolished GXYLT1^S212*^-induced metastasis and the mutation site may serve as a therapy indicator, further studies are required to justify the potential of ERK1/2 inhibitors to treat GXYLT1-mutant CRC.

In conclusion, this study provides insights into the genomic landscape of Chinese patients with CRC and identified new recurrent alterations in GXYLT1 not previously described in CRC. In addition, we found that the gain-of-function GXYLT1^S212*^ mutation showed a stronger capability to promote CRC metastasis than WT GXYLT1 by activating MAPK signaling. Furthermore, these findings suggest that GXYLT1^S212*^ could be used as a potential indicator for therapies targeting the MAPK pathway.

## Supplementary information

supplementary materials and methods

supplementary figure and table legends

supplementary figures

supplementary tables

Original western blots

Authors contribution statement

Reproducibility Checklist

## Data Availability

WES raw sequencing data generated during the current study have been submitted to the Sequence Read Archive (SRA) with BioProject ID: PRJNA745011 (http://submit.ncbi.nlm.nih.gov/). All other data generated during this study are included in this published article (and its supplementary information files).
