# Supplementary material for: A stop-gain mutation in GXYLT1 promotes metastasis of colorectal cancer via the MAPK pathway
Source: Cell Death Dis. 2022 Apr 22;13(4):395. doi: 10.1038/s41419-022-04844-3 (PMC9033806; doi:10.1038/s41419-022-04844-3)
Supplement: Supplementary file 1 — supplementary materials and methods [file 41419_2022_4844_MOESM1_ESM.docx]

**A stop-gain mutation in GXYLT1 promotes metastasis of colorectal cancer via the MAPK pathway**

Lin Peng^1,†^, Min Zhao^2,†^, Tianqi Liu^1^, Jiangbo Chen^1^, Pin Gao^1^, Lei Chen^1^, Pu Xing^1^, Zaozao Wang^1^, Jiabo Di^1^, Qiang Xu^4^, Hong Qu^3,*^, Beihai Jiang^1,*^, Xiangqian Su^1,*^

^†^ contributed equally

^*^ Corresponding authors

**Supplementary materials and methods**

**DNA extraction**

DNA was extracted using a Maxwell® RSC Instrument (cat# AS4500, Promega) automated DNA extraction system, according to the manufacturer’s instructions. DNA was isolated from freshly frozen tissue and normal peripheral blood leucocytes using a Maxwell RSC Blood DNA Kit (cat# AS1400, Promega) and a Maxwell RSC Whole Blood DNA Kit (cat# AS1520, Promega), respectively. Pure high molecular weight genomic DNA samples were quality checked on agarose gels and quantified using a Qubit 3.0 (Thermo Fisher Scientific, Rockford, IL, USA).

**Genetic variant calling for sequencing data**

Raw data generated using HiSeqX were first evaluated based on calculated Phred Quality Scores (Q). Clean reads were generated for further analysis using the Q30 (base call accuracy of 99.9 %) standard as the cut-off and were mapped to the human genome reference assembly (hg19) using the Burrows-Wheeler Aligner (1). The mapped SAM files were converted to BAM files, and PCR repeat markers were removed using Picard (version 1.115, https://broadinstitute.github.io/picard/). The Genome Analysis Toolkit (2) was used for necessary local realignment (GATK realignment) and quality score adjustments (GATK recalibration). Based on these adjusted high-quality data, we used three variant calling tools to check potential somatic variants: GATK-UG (UnifiedGenotyper), MuTech (version 1.1.7) (3), and VarScan2 (version 2.3.9) (4). Highly reliable genetic mutations were confirmed using at least two of these methods. To obtain high-confidence tumor-specific mutations, we removed genetic mutations that appeared in the normal blood samples of the same patient. The mutations identified in cancer samples were further annotated with the genomic location using Maftools (5). The overall features of insertions and deletions were calculated separately based on their unique chromosomal location and differences to the reference genome.

**Variant annotation and driver mutation ranking**

To identify potential Chinese population-specific driver mutations, we annotated all tumor-specific variants using snpEff (version 4.2) (6) and utilised the IntOGen-mutation pipeline including the two modules, OncodriveFM and OncodriveCLUST (7), to identify positively mutated genes during tumor clone evolution. In particular, OncodriveFM was used to identify whether a gene was statistically accumulating more mutations with a high functional impact (FM bias), whereas OncodriveCLUST identified genes whose mutations tend to cluster within the protein sequence.

**Droplet digital PCR (ddPCR)**

ddPCR was performed using a Naica Crystal Digital PCR System (Stilla Technologies, France). A 40× custom genotyping primer and probe mix were designed for GXYLT1^S212*^ using the sequence shown in Supplementary Table 6. Each 25 μL dPCR reaction mixture contained 5 μL PerfeCTa Muliiplex qPCR ToughMix 5× (Quanta Biosciences, Gaithersburg, Montgomery, USA), 2.5 μL fluorescein (1 μM), 0.625 μL TaqMan SNP Genotyping Assays 40× (cat #4332077, Thermo Fisher Scientific), 0.2 μg DNA, and nuclease-free water. The mixture was then loaded onto a Sapphire Chip (Stilla Technologies, Villejuif, France) and dPCR was performed according to the manufacturer’s protocol as follows: hot start at 95 °C for 5 min, followed by 45 cycles of 95 °C for 15 s and 60 °C for 1 min. Data were analyzed using a Crystal Reader (Stilla Technologies).

**Plasmid, siRNA, and lentivirus transfection**

The cDNAs of full-length GXYLT1 and mutant GXYLT1^S212*^ were cloned into the pCMV-3Tag-1A plasmid and Ubi-MCS-SV40-firefly_Luciferase-IRES-Puromycin lentiviral vector (Shanghai Genechem Co., Ltd. China), respectively. ON-target plus SMARTpool GXYLT1 small inhibitor RNA (siRNA) was purchased from Dharmacon (Cat #L-026987-02-0005, USA). CRC cells were transiently transfected with plasmids or siRNAs using Lipofectamine™ 2000 (Invitrogen, Grand Island, NY, USA) according to the manufacturer’s protocol. For lentiviral transfection, HCT116 cells were infected with the indicated lentiviral vectors and selected with 2 μg/mL puromycin for two weeks.

**Quantitative real-time PCR (qRT-PCR)**

Total RNA was extracted from CRC cells using TRIzol reagent (Cat #15596018, Thermo Fisher). cDNA was synthesised from total RNA using a reverse transcription kit (Promega, Madison, WI, USA). qRT-PCR was performed on an ABI 7500 Real-time PCR System (Life Technologies, Carlsbad, CA, USA) using SYBR Green Real-time PCR Master Mix (Cat # QPK-201, Toyobo Co., Ltd., Osaka, Japan). The primer sequences of qRT-PCR were: GXYLT1 Forward: GGCAGACTTGACAACTGGTCA, Reverse: CAGGATTAACGGCAAGAACAAT; GAPDH Forward: TGCACCACCAACTGCTTAGC, Reverse: GGCATGGACTGTGGTCATGAG. GXYLT1 mRNA expression was normalised to the internal control GAPDH and analyzed using the 2^–ΔΔCt^ method.

**Western blot**

Cells were harvested and lysed in RIPA buffer containing protease inhibitors. Protein concentration was quantified using a Bradford assay kit (Cat #5000205, Bio-Rad, Hercules, CA, USA). Equal amounts of proteins from different groups were separated using SDS-PAGE and transferred to polyvinylidene fluoride (PVDF) membranes (Millipore). After blocking with 5 % fat-free milk, the membranes were incubated with the primary and secondary antibodies listed in Supplementary Table 7. Proteins were visualised by chemiluminescence using an ECL Western blot detection kit (Cat #WBKLS0100, Millipore) and Amersham Imager 600 (GE Healthcare, Chicago, IL), then quantified using ImageJ software (NIH, USA).

**CCK8 assays**

After transfection with the indicated plasmids and siRNAs, 2000 cells were seeded into 96-well plates per well with 100 μL of medium. Cell viability was detected using a CCK-8 kit (#CK04, Dojindo, Japan) according to the manufacturer’s instructions. Absorbance was measured at 450 nm using a microplate reader (Bio-Rad, Hercules, CA, USA).

**Colony formation assay**

Cells transfected with plasmids or siRNAs were seeded into 6-well plates at a density of 1000 cells/well, with the culture medium replaced every three days. After incubation for 14 days, cell colonies were washed with PBS, fixed with ice-cold methanol, stained with 0.01 % crystal violet, and counted.

**Transwell migration and invasion assays**

Cell migration assays were performed using a Boyden chamber containing an 8.0 µm polycarbonate membrane (Cat #3422, Corning Costar, Cambridge, MA). Cells in 150 μL serum-free culture medium were placed into the upper chamber, while the lower chamber was filled with 600 μL medium with 10 % FBS. After incubation for 24 h at 37 °C, migrated cells were fixed with ice-cold methanol and stained with 0.5% crystal violet. Cells on the lower surface of the membrane were imaged in three randomly selected fields and counted under a microscope. Cell invasion assay was similarly performed except that the upper chamber was pre-covered with Matrigel matrix (Cat #356234, Corning, NY, USA).

**Wound healing assays**

CRC cells transfected with the indicated plasmids or siRNAs were seeded into 6-well plates and cultured to 90 % confluence. A scratch was made in the cell monolayer using a sterile 200 µL pipette tip and the cells were then washed with PBS and incubated in a serum-free culture medium. Images were obtained from three individual scratches at 0 and 48 h. The distance between the two edges was quantified using ImageJ software (NIH).

**References**

1. Zhang L, Liu C, Dong S. PipeMEM: A Framework to Speed Up BWA-MEM in Spark with Low Overhead. *Genes (Basel)* **10**, 886 (2019).
2. McKenna A, Hanna M, Banks E, Sivachenko A, Cibulskis K, Kernytsky A, et al. The Genome Analysis Toolkit: a MapReduce framework for analyzing next-generation DNA sequencing data. *Genome Res* **20**, 1297-303 (2010).
3. Cibulskis K, Lawrence M, Carter S, Sivachenko A, Jaffe D, Sougnez C, et al. Sensitive detection of somatic point mutations in impure and heterogeneous cancer samples. *Nat Biotechnol* **31**, 213-9 (2013).
4. Koboldt D, Zhang Q, Larson D, Shen D, McLellan M, Lin L, et al. VarScan 2: somatic mutation and copy number alteration discovery in cancer by exome sequencing. *Genome Res* **22**, 568-76 (2012).
5. Mayakonda A, Lin D, Assenov Y, Plass C, Koeffler H. Maftools: efficient and comprehensive analysis of somatic variants in cancer. *Genome Res* **28**, 1747-56 (2018).
6. Cingolani P, Platts A, Wang le L, Coon M, Nguyen T, Wang L, et al. A program for annotating and predicting the effects of single nucleotide polymorphisms, SnpEff: SNPs in the genome of Drosophila melanogaster strain w1118; iso-2; iso-3. *Fly (Austin)* **6**, 80-92 (2012).
7. Gonzalez-Perez A, Perez-Llamas C, Deu-Pons J, Tamborero D, Schroeder M, Jene-Sanz A, et al. IntOGen-mutations identifies cancer drivers across tumor types. *Nat Methods* **10**, 1081-2 (2013).
