# Supplementary material for: A stop-gain mutation in GXYLT1 promotes metastasis of colorectal cancer via the MAPK pathway
Source: Cell Death Dis. 2022 Apr 22;13(4):395. doi: 10.1038/s41419-022-04844-3 (PMC9033806; doi:10.1038/s41419-022-04844-3)
Supplement: Supplementary file 2 — supplementary figure and table legends [file 41419_2022_4844_MOESM2_ESM.docx]

**A stop-gain mutation in GXYLT1 promotes metastasis of colorectal cancer via the MAPK pathway**

Lin Peng^1,†^, Min Zhao^2,†^, Tianqi Liu^1^, Jiangbo Chen^1^, Pin Gao^1^, Lei Chen^1^, Pu Xing^1^, Zaozao Wang^1^, Jiabo Di^1^, Qiang Xu^4^, Hong Qu^3,*^, Beihai Jiang^1,*^, Xiangqian Su^1,*^

^†^ contributed equally

^*^ Corresponding authors

**Supplementary figure legends**

**Supplementary Figure 1. Overview of the somatic mutations in 45 colorectal cancer (CRC) samples.** (a) Variant classification in the 45 CRC samples. (b) Variant types in the 45 CRC samples. (c) Overall distribution of transitions (Ti) and transversions (Tv) in the mutations identified in the 45 CRC samples. (d) Proportion of the six types of base substitutions detected in the 45 CRC samples.

**Supplementary Figure 2.** **Tumor mutation burden (TMB) distribution in our cohort and 33 TCGA datasets.**

**Supplementary Figure 3. Mutational signature in the 45 CRC samples.** (a) Relative contribution of the 30 known COSMIC mutational signatures in each patient. (b) Similarities between the 30 known COSMIC mutational signatures and the four mutational signatures identified in our cohort. Darker colors indicate a higher similarity.

**Supplementary Figure 4. High *GXYLT1* expression predicts poor prognosis in patients with CRC.** (a) Analysis of *GXYLT1* mRNA expression levels in CRC tumors and matched normal tissues using GEO datasets (GSE24550 and GSE9348). (b) Analysis of *GXYLT1* mRNA expression levels with respect to tumor stage using GEO datasets (GSE33193 and GSE28702). (c) Kaplan–Meier survival analysis of survival according to *GXYLT1* levels and CRC patient using GEO datasets (GSE38832 and GSE17538).

**Supplementary Figure 5. Detection of GXYLT1^S212*^ using droplet digital PCR in a representative CRC sample.** Two-dimensional Droplet Digital PCR plots showing the detection of the non-template control (NTC) (a), positive control (b), negative control (c), and a representative sample with *GXYLT1^S212*^* (d). FAM, mutant probe; VIC, wild-type probe. Positive dots are located above each threshold line.

**Supplementary Figure 6. GXYLT1 and GXYLT1^S212*^ expression in CRC cells.** (a, b) GXYLT1 mRNA and protein levels were determined in CRC cells by qRT-PCR and western blot analysis, respectively. (c) Overexpression of GXYLT1 and GXYLT1^S212*^ in LoVo and HCT116 cells was measured using Western blot by GXYLT1 antibody. (d) GXYLT1 and GXYLT1^S212*^ overexpression in LoVo and HCT116 cells was measured using qRT-PCR and western blot against flag. (e) GXYLT1 knockdown in RKO and SW480 cells was measured using qRT-PCR and western blot. Data are presented as the mean ± SD of at least three independent experiments.

**Supplementary Figure 7. Notch pathway inhibitor partially impaired metastasis induced by GXYLT1^S212*^.** (a, b) Transwell assays of the migration of transfected CRC cells treated with the Notch pathway inhibitor DAPT for 48 h. Graphs show quantification of migrated cells. (c, d) Western blot analysis of NICD and Hes1 protein levels. Bar graphs show quantification of NICD and Hes1 levels. Data are presented as the mean ± SD of at least three independent experiments. * *p* < 0.05, ** *p* < 0.01, ****p* < 0.001.

**Supplementary Figure 8. Pearson correlation analyses of the mRNA expression levels of EGFR, ERK2 and GXYLT1 in public CRC databases.** (a) Correlation of GXYLT1 and EGFR mRNA expression levels in CRC patients from TCGA. (b) Correlation of GXYLT1 and ERK2 mRNA expression levels in CRC patients from TCGA. (c) Correlation of GXYLT1 and EGFR mRNA expression levels in CRC patients from the GSE24550 dataset. (d) Correlation of GXYLT1 and ERK2 mRNA expression levels in CRC patients from the GSE24550 dataset.

**Supplementary Figure 9.** **GXYLT1^S212*^ promote CRC cells migration and invasion in vitro while GXYLT1^Y264*^ have no significant effect.** (a, b) Overexpression of GXYLT1, GXYLT1^S212*^ and GXYLT1^Y264*^ in LoVo cells was measured using Western blot by GXYLT1 antibody (a) and flag antibody (b). (c) Transwell migration assay of transfected CRC cells. Graphs show quantification of migrated cells. ****p* < 0.001.

**Supplementary Figure 10. Pearson correlation analyses of the mRNA expression levels of GXYLT1 and GXYLT2 in GEO datasets.**

**Supplementary Tables**

**Supplementary Table 1.** Patient characteristics

**Supplementary Table 2.** Detailed clinical information for the 45 CRC samples

**Supplementary Table 3.** Somatic mutations found in exomes of the 45 CRC samples

**Supplementary Table 4.** Genes arranged by alteration frequency identified in the 45 CRC samples

**Supplementary Table 5.** Incidence of mouse liver metastasis

**Supplementary Table 6.** Primers and probes for ddPCR GXYLT1^S212*^ (c.635G>T; p.Ser212*)

**Supplementary Table 7.** Antibodies for western blot
