# Supplementary material for: A stop-gain mutation in GXYLT1 promotes metastasis of colorectal cancer via the MAPK pathway
Source: Cell Death Dis. 2022 Apr 22;13(4):395. doi: 10.1038/s41419-022-04844-3 (PMC9033806; doi:10.1038/s41419-022-04844-3)

Figure 4a

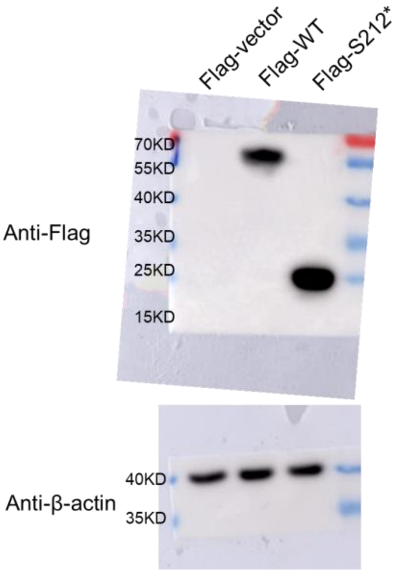

Figure 5a

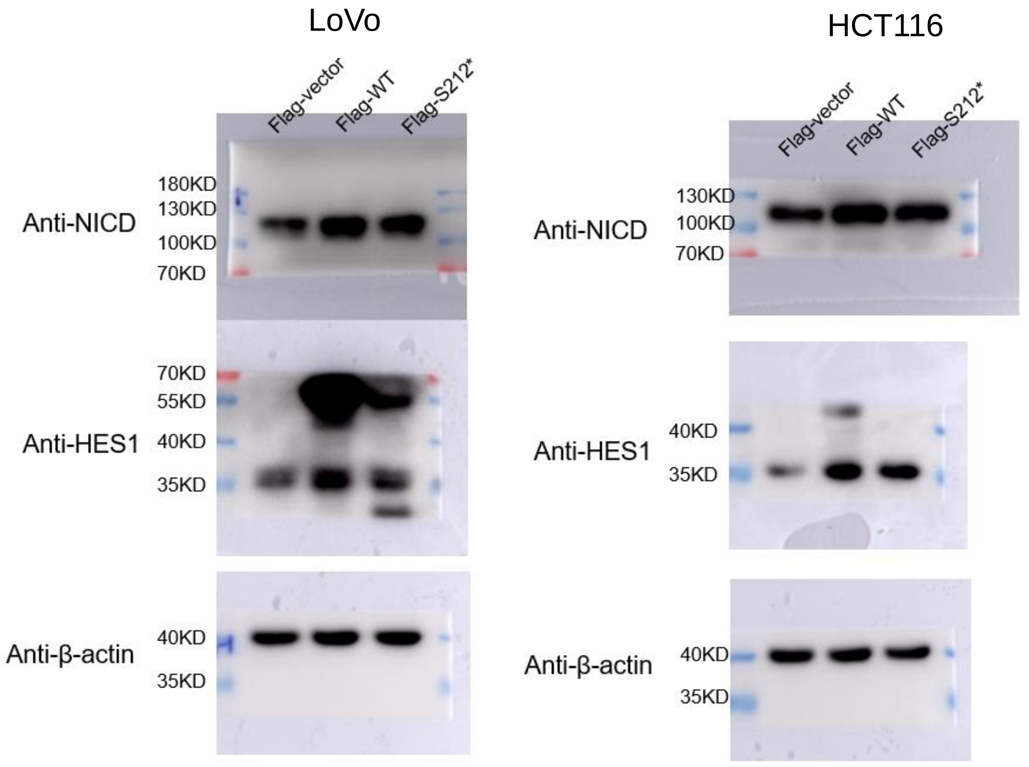

Figure 5b

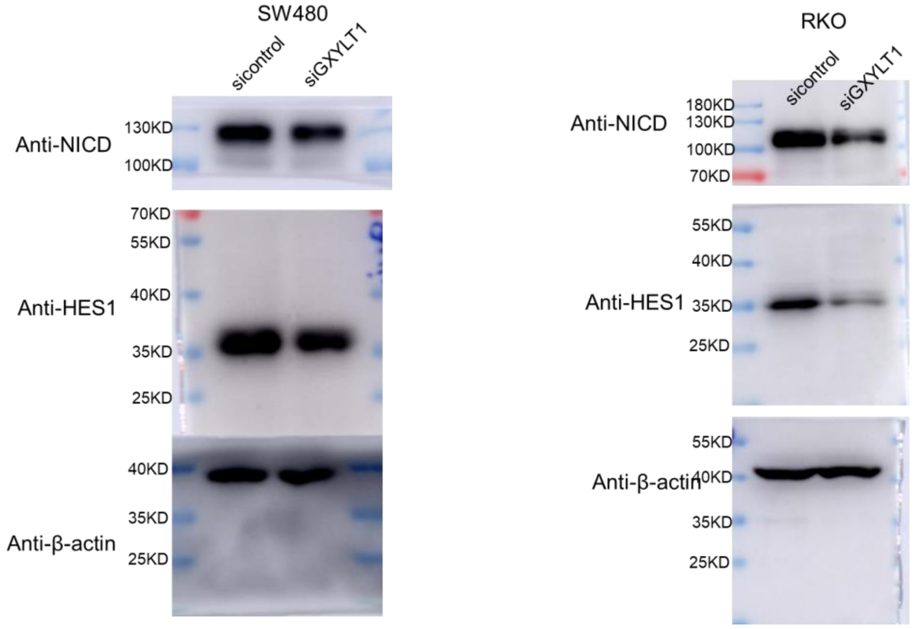

Figure 5e

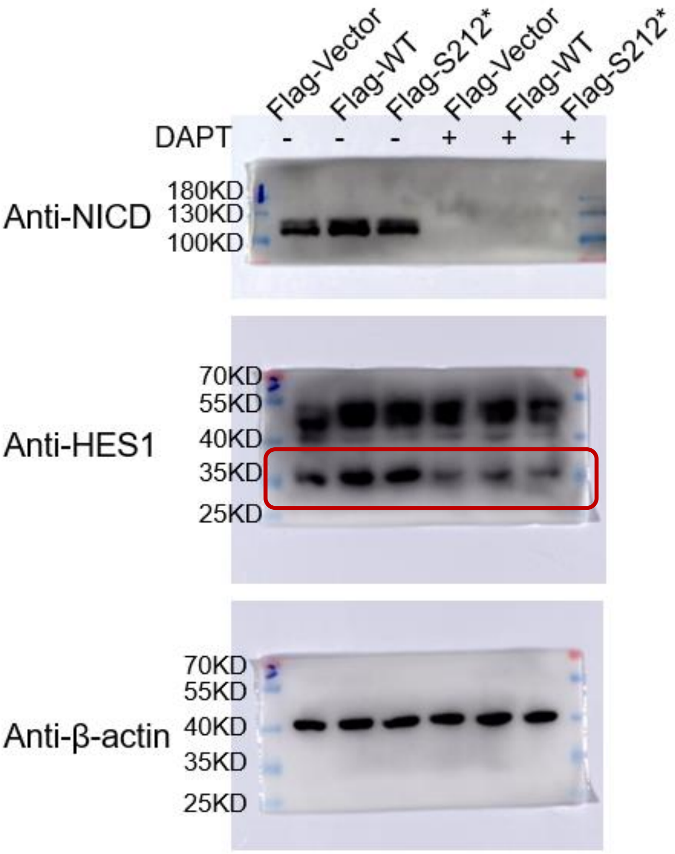

Figure 5f

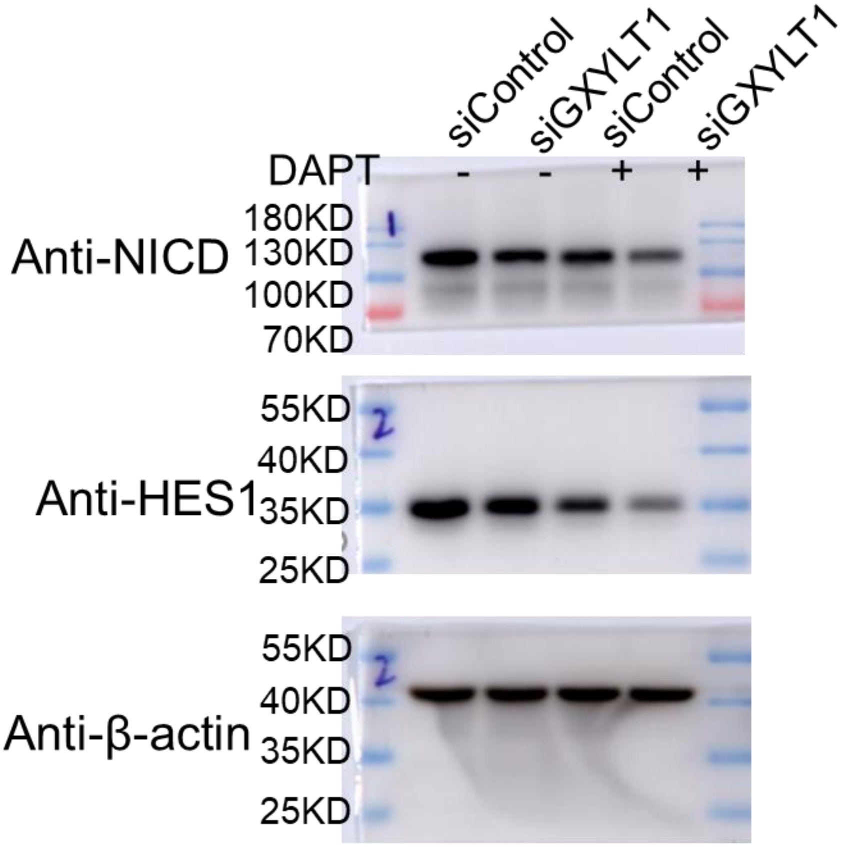

siControl
siGXYLT1
siControl
siGXYLT1
DAPT
-
-
+
+
Anti-NICD
180KD
130KD
100KD
70KD
Anti-HES1
55KD
40KD
35KD
25KD
Anti-β-actin
55KD
40KD
35KD
25KD

Figure 6b

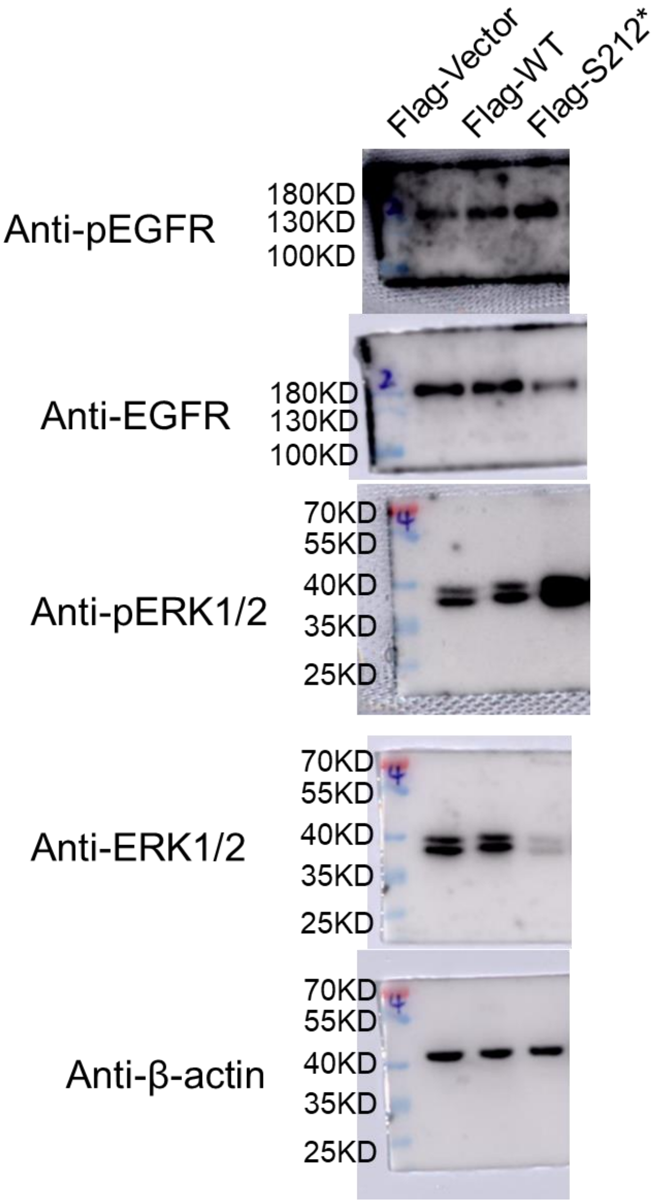

Flag-Vector
Flag-WT
Flag-S212*
Anti-pEGFR
180KD
130KD
100KD
Anti-EGFR
180KD
130KD
100KD
Anti-pERK1/2
70KD
55KD
40KD
35KD
25KD
Anti-ERK1/2
70KD
55KD
40KD
35KD
25KD
Anti-β-actin
70KD
55KD
40KD
35KD
25KD

Figure 6c

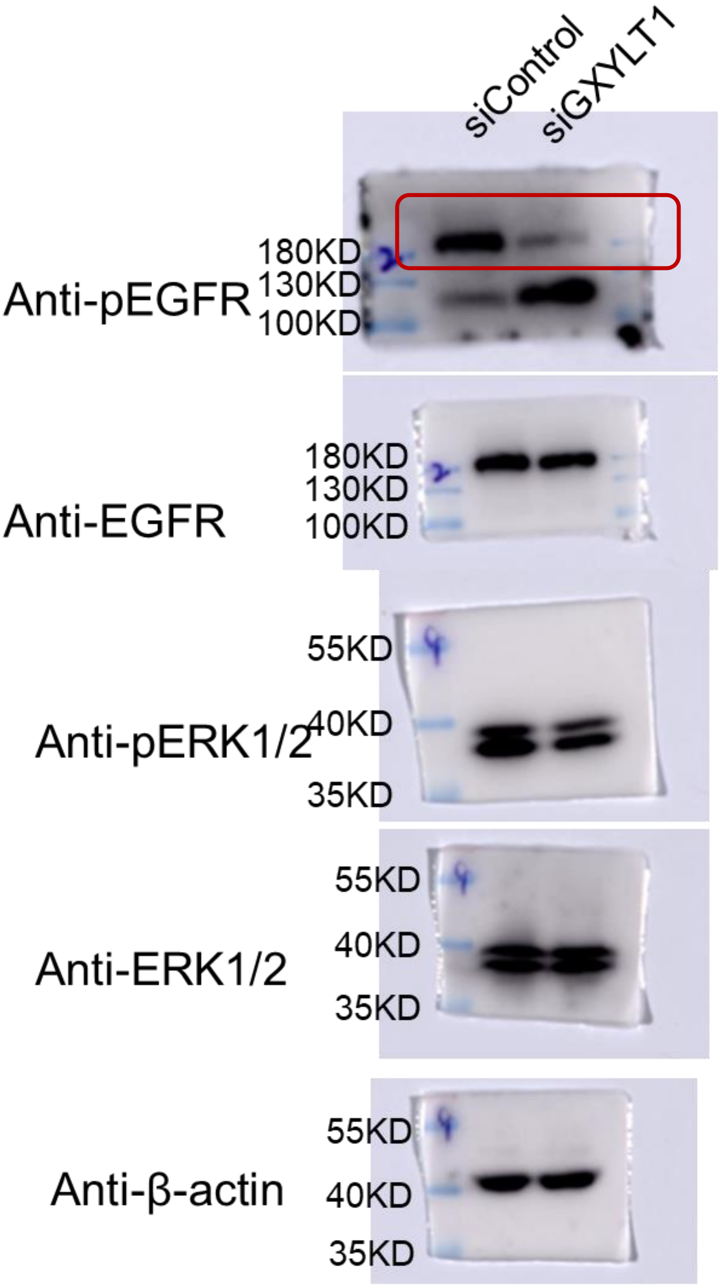
siControl
siGXYLT1
Anti-pEGFR
180KD
130KD
100KD
Anti-EGFR
180KD
130KD
100KD
Anti-pERK1/2
55KD
40KD
35KD
Anti-ERK1/2
55KD
40KD
35KD
Anti-β-actin
55KD
40KD
35KD

Figure 6d

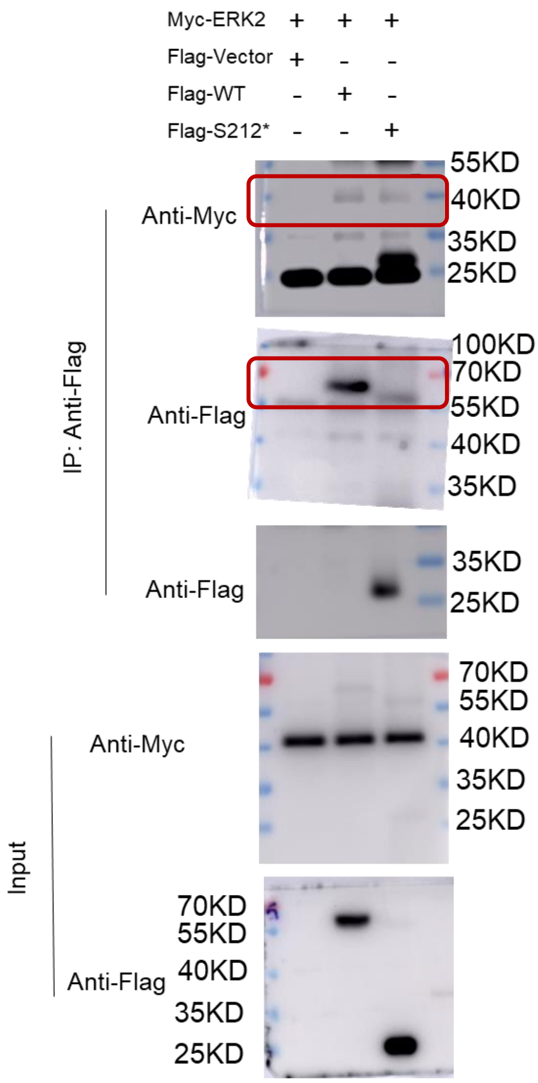

Myc-ERK2 + + +
Flag-Vector + - -
Flag-WT - + -
Flag-S212* - - +
IP: Anti-Flag
Anti-Myc
55KD
40KD
35KD
25KD
Anti-Flag
100KD
70KD
55KD
40KD
35KD
Anti-Flag
35KD
25KD
Input
Anti-Myc
70KD
55KD
40KD
35KD
25KD
Anti-Flag
70KD
55KD
40KD
35KD
25KD

Figure 6e

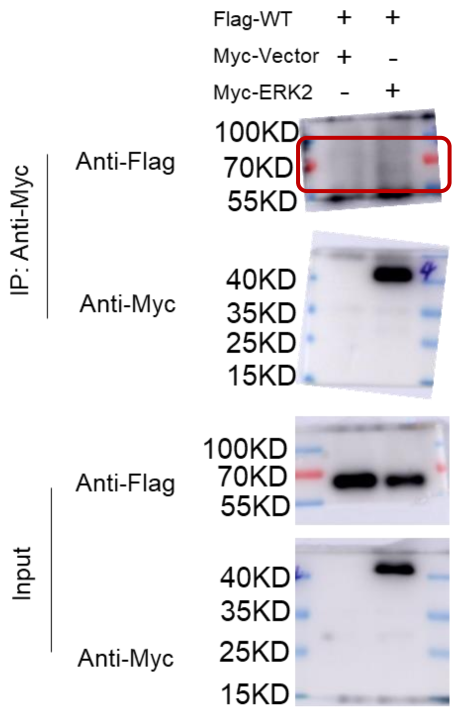

Figure 6f

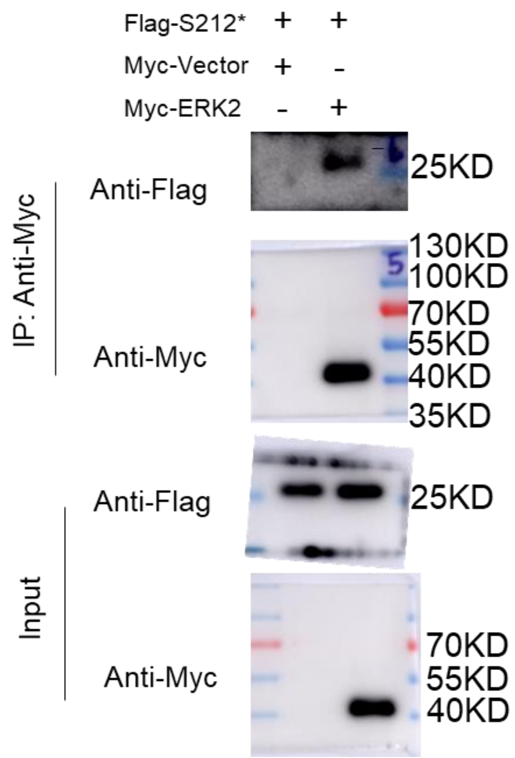

Figure 6i

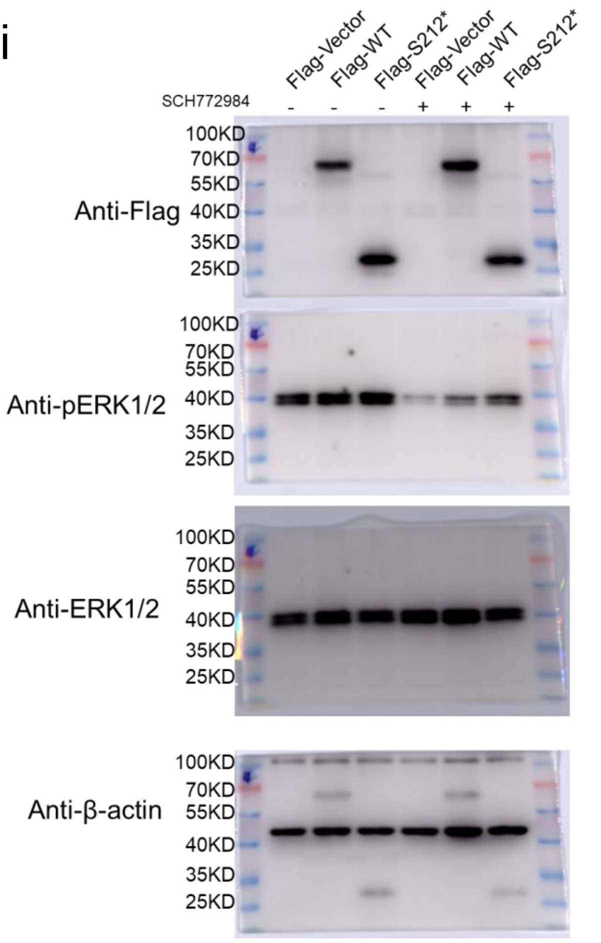

Figure 6j

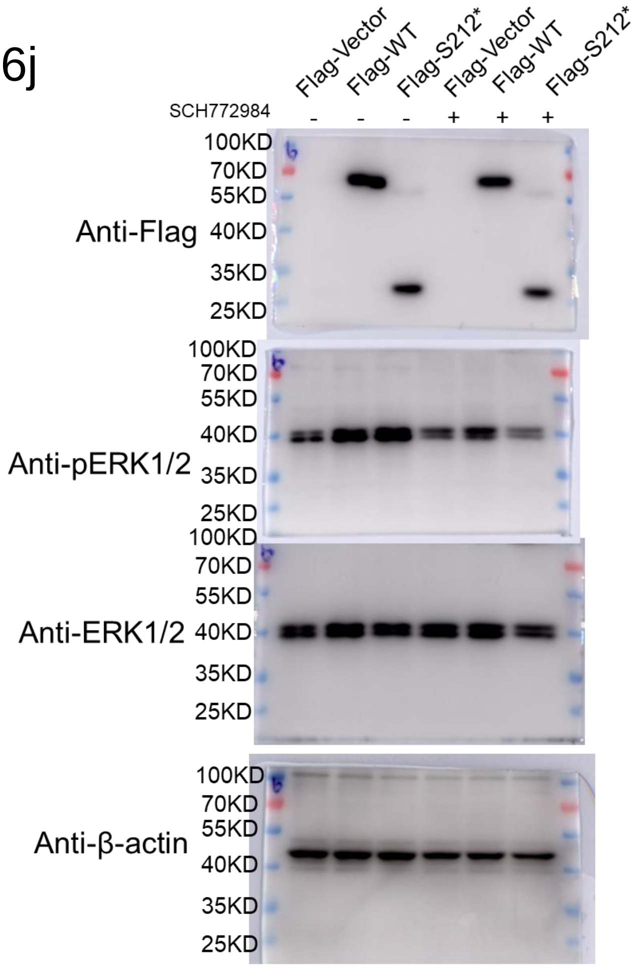

Figure 7a

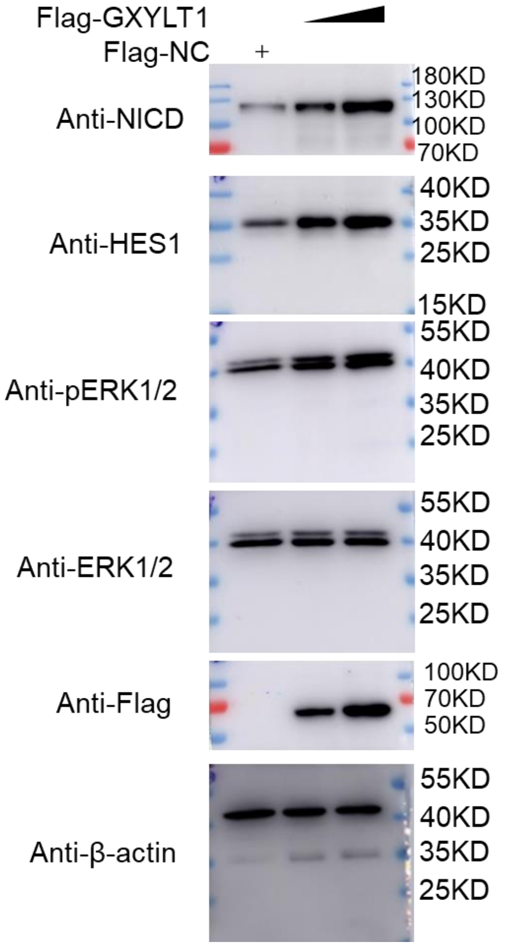
Flag-GXYLT1
Flag-NC
+
Anti-NICD
180KD
130KD
100KD
70KD
Anti-HES1
40KD
35KD
25KD
15KD
Anti-pERK1/2
55KD
40KD
35KD
25KD
Anti-ERK1/2
55KD
40KD
35KD
25KD
Anti-Flag
100KD
70KD
50KD
Anti-β-actin
55KD
40KD
35KD
25KD

Figure 7b

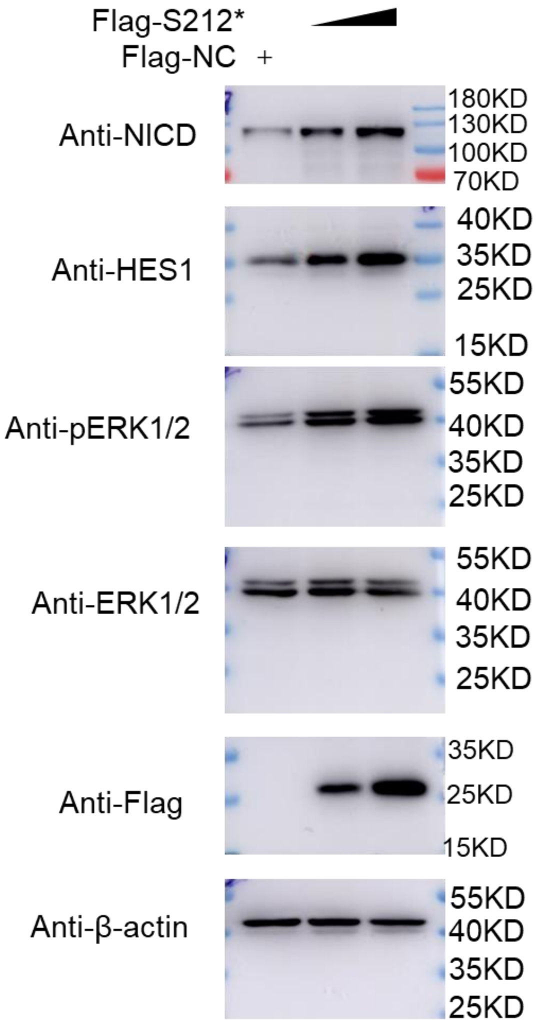
Flag-S212*
Flag-NC +
Anti-NICD
180KD
130KD
100KD
70KD
Anti-HES1
40KD
35KD
25KD
15KD
Anti-pERK1/2
55KD
40KD
35KD
25KD
Anti-ERK1/2
55KD
40KD
35KD
25KD
Anti-Flag
35KD
25KD
15KD
Anti-β-actin
55KD
40KD
35KD
25KD

Figure 7c

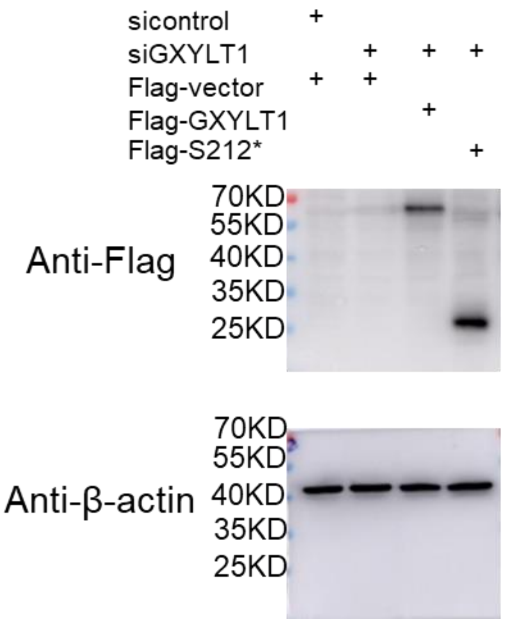

Figure 7e

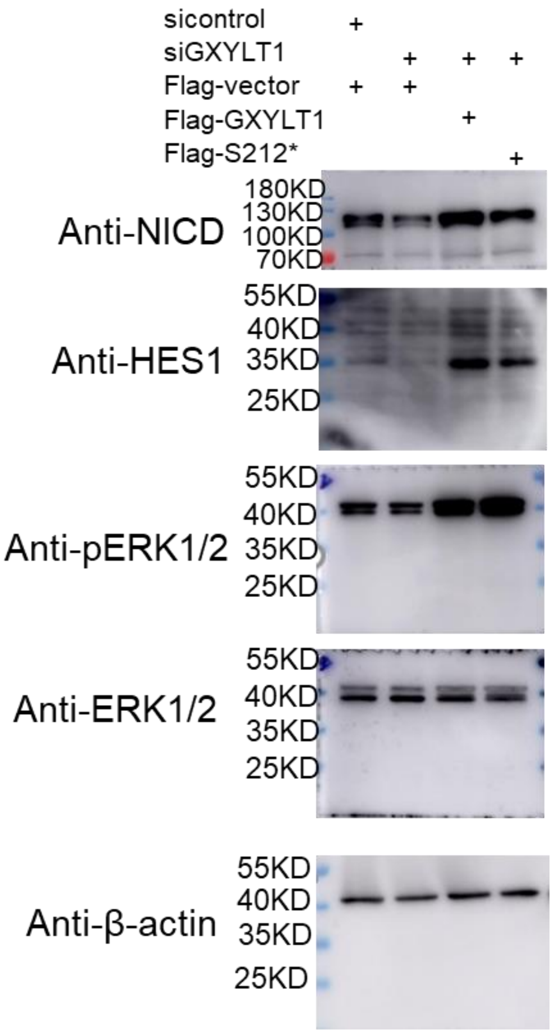

Supplementary Figure 6b

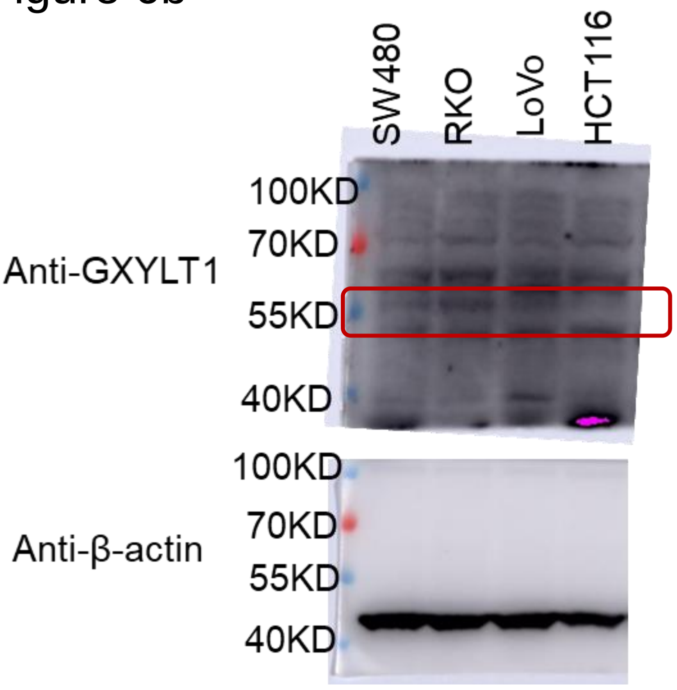

Supplementary Figure 6c

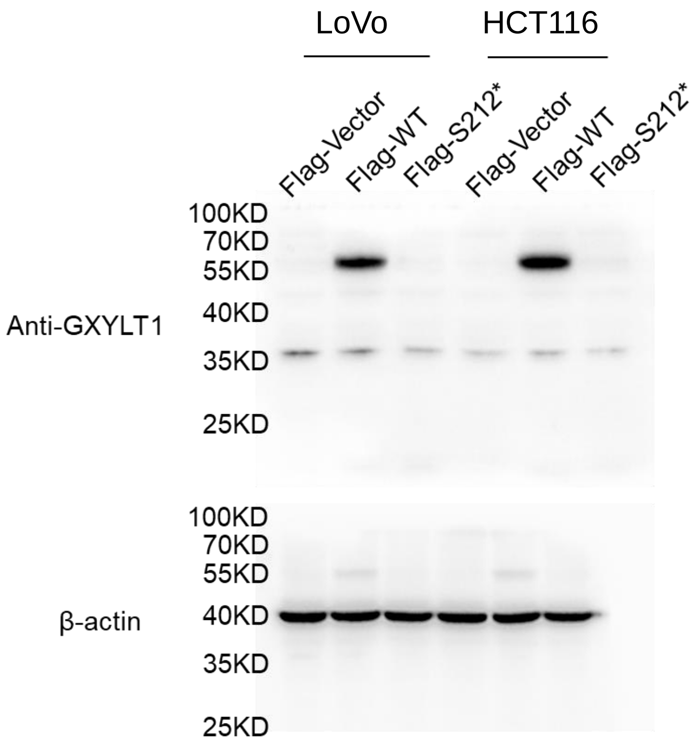

Supplementary Figure 6d

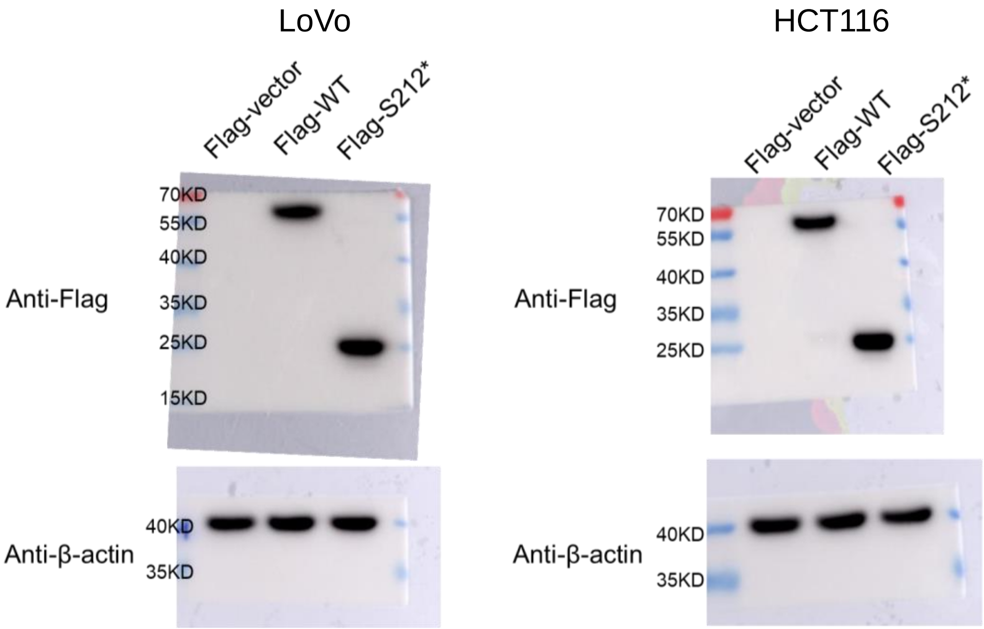

Supplementary Figure 6e

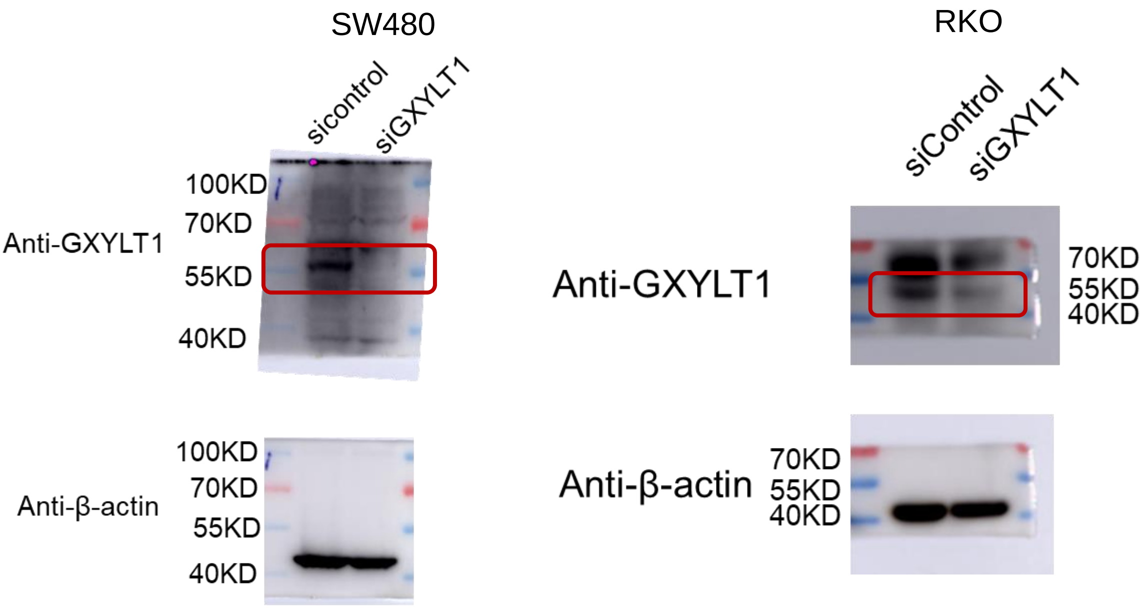

Supplementary Figure 7c

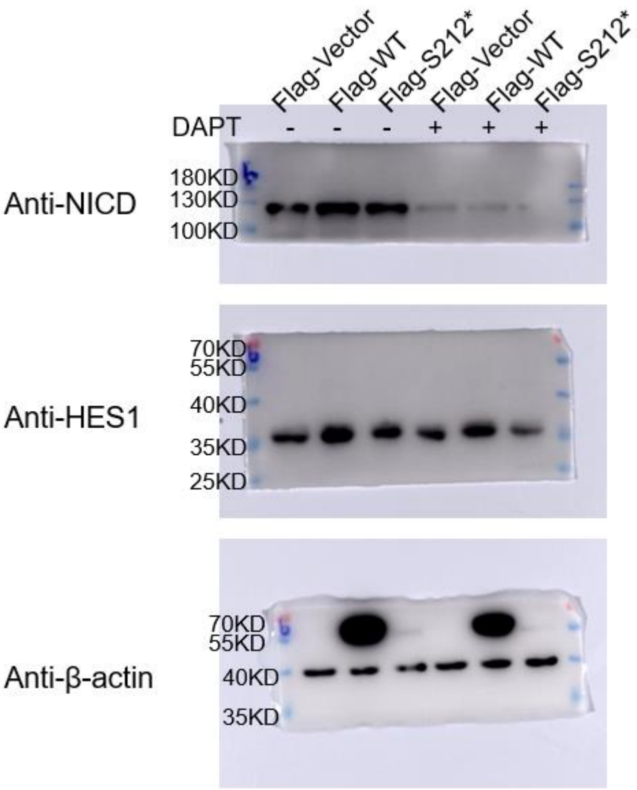

Supplementary Figure 7d

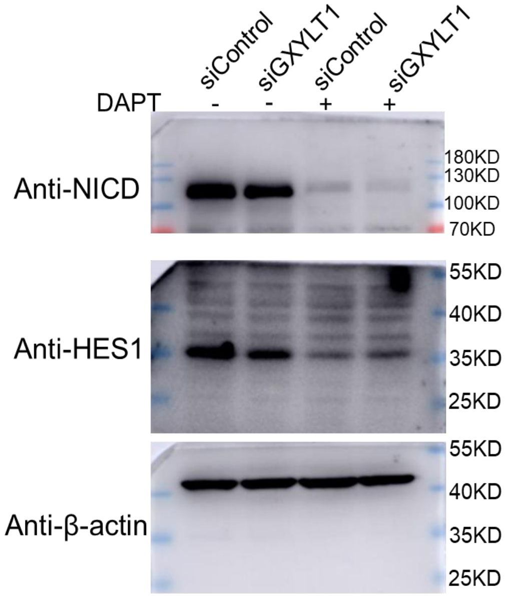

Supplementary Figure 9a

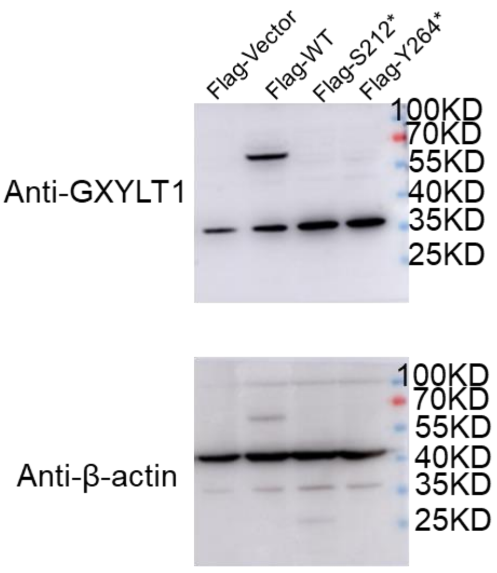

Supplementary Figure 9b

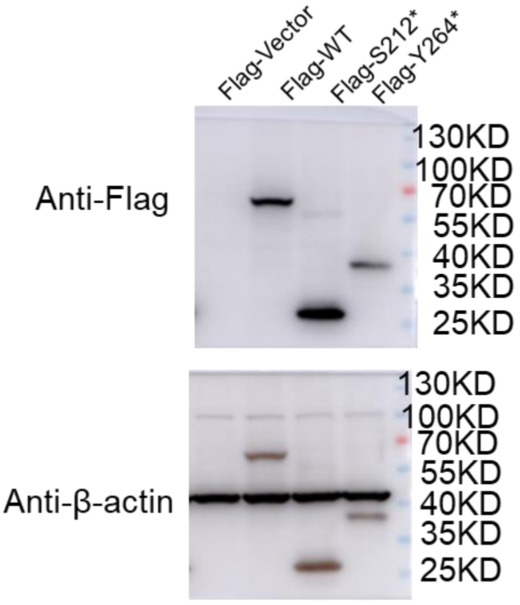

Supplement: Supplementary file 5 — Original western blots [file 41419_2022_4844_MOESM5_ESM.pdf]
