# Supplementary material for: A stop-gain mutation in GXYLT1 promotes metastasis of colorectal cancer via the MAPK pathway
Source: Cell Death Dis. 2022 Apr 22;13(4):395. doi: 10.1038/s41419-022-04844-3 (PMC9033806; doi:10.1038/s41419-022-04844-3)
Supplement: Supplementary file 6 — Authors contribution statement [file 41419_2022_4844_MOESM6_ESM.docx]

**A stop-gain mutation in GXYLT1 promotes metastasis of colorectal cancer via the MAPK pathway**

Lin Peng^1,†^, Min Zhao^2,†^, Tianqi Liu^1^, Jiangbo Chen^1^, Pin Gao^1^, Lei Chen^1^, Pu Xing^1^, Zaozao Wang^1^, Jiabo Di^1^, Qiang Xu^4^, Hong Qu^3,*^, Beihai Jiang^1,*^, Xiangqian Su^1,*^

^†^ contributed equally

^*^ Corresponding authors

**Author Contribution Statement**

HQ, BJ, and XS conceived and designed the study. LP, TL, JC, PG, and PX performed the experiments. MZ performed the bioinformatics data analysis. LC, ZW, JD, and QX contributed samples, reagents, materials, and comments on the manuscript. LP, MZ, and BJ wrote the manuscript. All authors read and approved the final manuscript.
